# The alarmones (p)ppGpp are part of the heat shock response of *Bacillus subtilis*

**DOI:** 10.1371/journal.pgen.1008275

**Published:** 2020-03-16

**Authors:** Heinrich Schäfer, Bertrand Beckert, Christian K. Frese, Wieland Steinchen, Aaron M. Nuss, Michael Beckstette, Ingo Hantke, Kristina Driller, Petra Sudzinová, Libor Krásný, Volkhard Kaever, Petra Dersch, Gert Bange, Daniel N. Wilson, Kürşad Turgay

**Affiliations:** 1 Institute of Microbiology, Leibniz Universität Hannover, Hannover, Germany; 2 Max Planck Unit for the Science of Pathogens, Berlin, Germany; 3 Institute for Biochemistry and Molecular Biology, University of Hamburg, Hamburg, Germany; 4 Philipps-University Marburg, Center for Synthetic Microbiology (SYNMIKRO) and Department of Chemistry, Marburg, Germany; 5 Department of Molecular Infection Biology, Helmholtz Centre for Infection Research, Braunschweig, Germany; 6 Institute of Microbiology, Czech Academy of Sciences, Prague, Czech Republic; 7 Hannover Medical School, Research Core Unit Metabolomics, Hannover, Germany; 8 Institute of Infectiology, University of Münster, Münster, Germany; University of Wisconsin-Madison, UNITED STATES

## Abstract

*Bacillus subtilis* cells are well suited to study how bacteria sense and adapt to proteotoxic stress such as heat, since temperature fluctuations are a major challenge to soil-dwelling bacteria. Here, we show that the alarmones (p)ppGpp, well known second messengers of nutrient starvation, are also involved in the heat stress response as well as the development of thermo-resistance. Upon heat-shock, intracellular levels of (p)ppGpp rise in a rapid but transient manner. The heat-induced (p)ppGpp is primarily produced by the ribosome-associated alarmone synthetase Rel, while the small alarmone synthetases RelP and RelQ seem not to be involved. Furthermore, our study shows that the generated (p)ppGpp pulse primarily acts at the level of translation, and only specific genes are regulated at the transcriptional level. These include the down-regulation of some translation-related genes and the up-regulation of *hpf*, encoding the ribosome-protecting hibernation-promoting factor. In addition, the alarmones appear to interact with the activity of the stress transcription factor Spx during heat stress. Taken together, our study suggests that (p)ppGpp modulates the translational capacity at elevated temperatures and thereby allows *B*. *subtilis* cells to respond to proteotoxic stress, not only by raising the cellular repair capacity, but also by decreasing translation to concurrently reduce the protein load on the cellular protein quality control system.

## Author summary

We observed that the second messenger (p)ppGpp, known to be synthesized by the ribosome-associated Rel synthetase upon nutrient starvation during the stringent response, is also intricately involved in the stress response of *B*. *subtilis* cells and can act as a pleiotropic regulator during the adaptation to heat stress. (p)ppGpp can slow down and modulate translation and is, together with the transcriptional stress regulator Spx, partially involved in the transcriptional down-regulation of the translation machinery. The stress-induced elevation of cellular (p)ppGpp levels confers increased stress tolerance and facilitates an improved protein homeostasis by modulating translation and reducing the load on the protein quality control system.

## Introduction

Bacteria have evolved complex and diverse regulatory networks to sense and respond to changes in the environment, which can include physical stresses or nutrient limitation [1]. The universally conserved protein quality control system comprises a conserved set of chaperones and proteases that monitor and maintain protein homeostasis. Various physical stresses, such as heat stress, favor the unfolding and aggregation of cellular proteins, which can be sensed by heat shock response systems, allowing an appropriate cellular stress response. The response to such protein unfolding stresses includes the induction of the expression of genes encoding chaperones and proteases of the quality control system, also known as heat shock proteins, and is usually very fast (less than 2–5 min) [2–7].

Interestingly, in *B*. *subtilis* cells, a short exposure to a raised but non-lethal temperature induces thermotolerance, an acquired resistance to otherwise lethal temperatures [8,9]. Investigating the adaptation to such adverse conditions, also known as priming, allows the molecular mechanisms and interplay of the various cellular processes involved in the cellular stress and heat shock response to be studied [8,9]. In *B*. *subtilis*, the heat shock response is orchestrated by multiple transcriptional regulators, including the heat-sensitive repressors HrcA & CtsR, which control the expression of the protein quality control system and other general stress genes [10–13]. The general stress response, activated by the alternative sigma factor σ^B^, is controlled by a complex regulatory network that integrates diverse stress and starvation signals, including heat [14]. In addition, Spx is a central regulator of the heat and thiol stress response, which is important for the development of thermotolerance. Spx activates the expression of many genes of the heat shock response, including *clpX*, *htpG* and genes of the oxidative and thiol stress response such as thioredoxin [9,15–18]. Interestingly, Spx can also mediate the inhibition of cell growth by the concurrent transcriptional down-regulation of many translation-related genes [17].

Another fast-acting bacterial stress response system is the stringent response (SR), which is mediated by the second messenger alarmones (p)ppGpp [19]. The synthesis and hydrolysis of (p)ppGpp is catalyzed by RelA/SpoT homologs (RSH) which contain N-terminal synthetase and hydrolase domains (bifunctional Rel or SpoT subgroup), or an active synthetase and an inactive hydrolase domain (RelA subgroup) together with additional regulatory domains at the C-terminus [20]. RSH can therefore direct both synthesis and, in the case of Rel, hydrolysis of (p)ppGpp. The enzyme activity of RelA or Rel is stimulated by association with uncharged tRNAs and the ribosome, thereby mediating (p)ppGpp synthesis upon amino acid starvation [21–25]. In addition to this long multi-domain RSH form, monofunctional small alarmone synthetases (SAS) or small alarmone hydrolases (SAH) with single synthetase or hydrolase domains are present in many bacteria [26]. In *B*. *subtilis*, alarmone levels are controlled by Rel (often referred to as RelA), a bifunctional, RSH-type synthetase/hydrolase as well as two SAS proteins RelP (SasA, YwaC) and RelQ (SasB, YjbM) [27–29].

The synthesis and hydrolysis of (p)ppGpp allows the activation or repression of different cellular pathways by modulating various enzyme activities involved in GTP homeostasis, replication, transcription and translation, not only in response to amino acid starvation, but also to various other signals or stresses. It was observed for different bacteria that additional and diverse starvation or stress signals can activate the SR via interacting proteins or metabolites that bind and modulate the activity of RSH-type enzymes, or by transcriptional or post-translational regulation of monofunctional SAS [30,31]. *B*. *subtilis* and related Firmicutes lack a DksA homolog and a direct binding site for (p)ppGpp on RNA polymerase (RNAP) which mediate positive and negative stringent regulation in *E*. *coli* and other proteobacteria. Instead, in *B*. *subtilis* (p)ppGpp can exert transcriptional regulation via a drop in GTP levels caused by the direct inhibition of multiple enzymes of the GTP synthesis pathway [32,33]. Thus, transcription of ribosomal RNA (rRNA) and ribosomal protein (r-protein) genes from promoters that initiate transcription with GTP is strongly reduced, while in turn promoters that initiate with ATP are activated [34,35]. In addition, the global regulator and repressor CodY is activated by GTP via an allosteric binding site, and therefore amino acid biosynthesis genes and other pathways are de-repressed upon a drop of the cellular GTP level during the SR [36,37]. Beyond its impact on transcription, (p)ppGpp can modulate ribosome assembly, translation initiation and elongation by binding, for example, to the translation initiation factor IF-2 and other ribosome-associated GTPases [38–44]. With its ability to inhibit translation and growth, the SR was also implicated in persister cell formation and development of antibiotic tolerance [45]. In addition, (p)ppGpp is required for virulence as well as survival of pathogens during infection [19,46].

During exposure to heat and oxidative stress, we and others previously observed in *B*. *subtilis* a pronounced down-regulation of rRNA and r-protein genes that resembled the pattern of the SR [16–18,47]. Thus, we hypothesized that the alarmone (p)ppGpp and the SR-like response could be part of the heat shock response of *B*. *subtilis*. Therefore, we investigated the role of the SR and its intricate and mutual involvement with the cellular stress response under various proteotoxic stress conditions, including various heat shock conditions [9,48].

Consistent with our hypothesis, we could demonstrate that the cellular level of (p)ppGpp was increased upon heat shock, as well as upon salt and oxidative stress. In addition, raised alarmone levels conferred increased stress tolerance and a (p)ppGpp^0^ strain appeared more stress sensitive. The presence of the bifunctional Rel was necessary and sufficient for the observed stress induced increase of (p)ppGpp. Overall (p)ppGpp appeared to play only a minor more complementary role for the heat mediated adjustments of transcription. However, we observed a prominent and instantaneous effect of the cellular alarmone (p)ppGpp levels on limiting and modulating translation by reducing the protein load on the quality control system during heat stress and concurrently allowing the expression of heat shock genes. Thereby the fast reallocation of cellular resources to raise the cellular repair capacity controlled by the other known regulators of the heat shock response could be facilitated.

## Results and discussion

### Regimes for monitoring of heat shock stress response in *B*. *subtilis*

**Fig 1:**
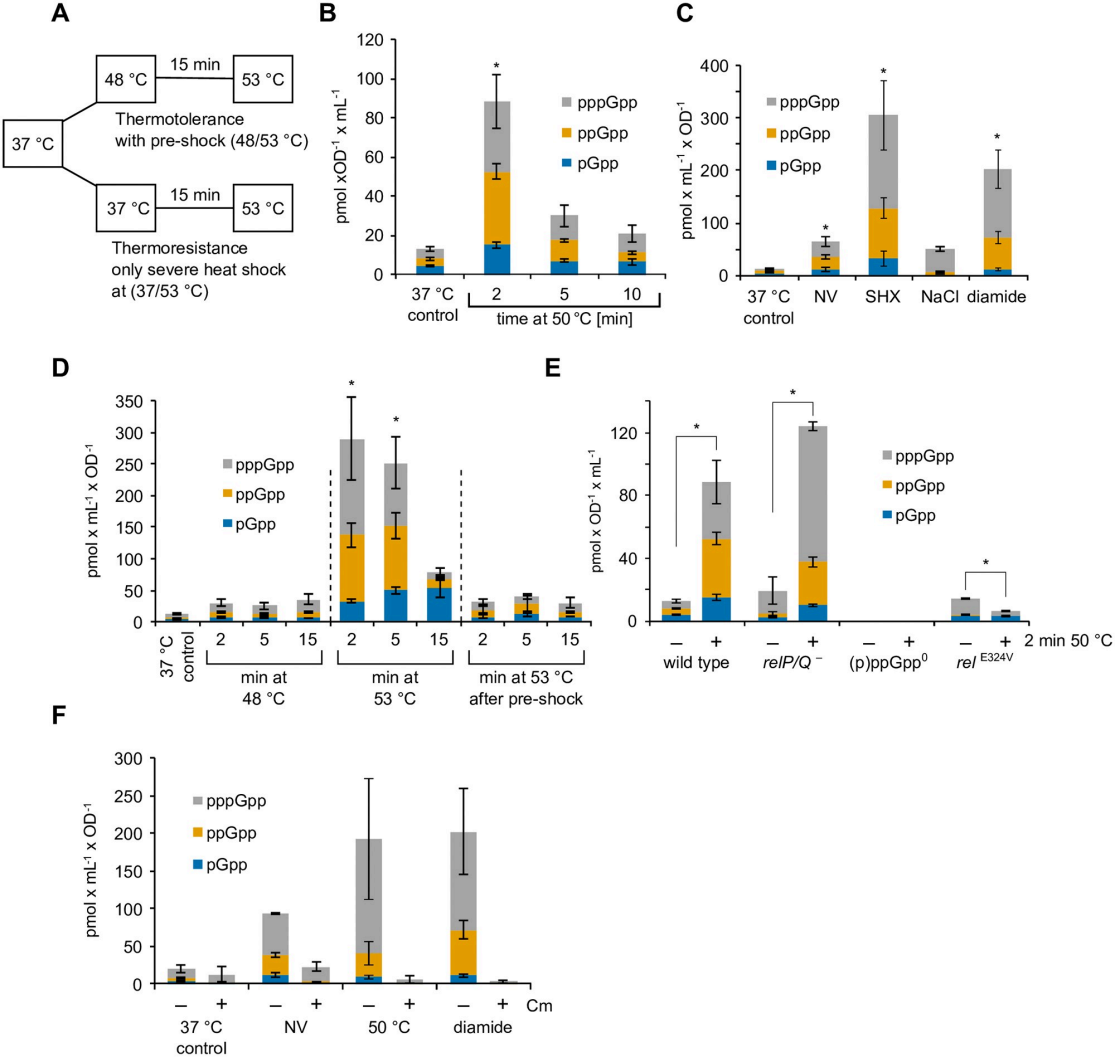
(p)ppGpp levels are increased by heat shock and stress. **(A)** Outline of the thermotolerance protocol. A culture of cells growing exponentially at 37 °C is divided and incubated at 48 °C or left at 37 °C. After 15 min, both cultures are shifted to 53 °C. **(B-F)** Levels of pGpp, ppGpp and pppGpp under different conditions. Asterisks (*) indicate significance (*p*_*adj*._ ≤ 0.05) of combined pGpp, ppGpp and pppGpp levels according to the Kruskal-Wallis and Dunn-Bonferroni test. **(B)** Cells were grown in minimal medium to OD_600_ of 0.4 and transferred to 50 °C. Means and and standard error of mean (SEM) of four independent experiments are shown. **(C)** Cells were grown in minimal medium to the mid-exponential phase (OD_600_ ~ 0.4) and treated with DL-norvaline (NV; 0.5 mg ml^-1^), serine hydroxamate (SHX; 5 μg ml^-1^), NaCl (6%) or diamide (0.5 mM) for 10 min. Means and SEM of three to four independent experiments are shown. **(D)** Wild type cells were grown at 37 °C and shifted to 48 °C for 15 min (pre-shock), then to 53 °C or directly to 53 °C. Samples were taken at 2, 5 and 15 min. Means and SEM of four independent experiments are shown. **(E)** Wild type cells or strains with mutations in (p)ppGpp synthetases (*relP/Q* ^-^: BHS204, *rel*^E324V^: BHS709; (p)ppGpp°: BHS214) were treated with or without heat shock at 50 °C for 2 min. Means and SEM of three to six independent experiments are shown. No alarmone peaks were detected in the (p)ppGpp° mutant (lower limit of quantification: 0.26 pmol x mL^-1^ x OD^-1^). Asterisks (*) indicate significant changes (*p* ≤ 0.05) of combined pGpp, ppGpp and pppGpp levels according to Welch’s *t*-test. **(F)** The influence of chloramphenicol on alarmone accumulation during stress. Cells were grown in minimal medium and treated with DL-norvaline (0.5 mg ml^-1^) for 10 min, heat shock at 50 °C for 2 min or diamide (1 mM) for 10 min. Chloramphenicol (Cm, 25 μg ml^-1^) was added at the same time to one part. Means and SEM of two independent experiments are shown.

In this study, we investigated the stress response of *B*. *subtilis* by application of different, but related, heat shock conditions: (i) growth and heat shock at 50 °C, a temperature that is non-lethal for *B*. *subtilis* but already induces a significant heat shock response with a raised expression of chaperones and proteases, (ii) resistance to severe heat shock by measuring the survival of exponentially growing cells exposed to a severe, lethal heat shock at 53 °C, which can also be considered a measure for thermoresistance (37/53 °C) (Fig 1A), and (iii) the development of thermotolerance by measuring the survival of exponentially growing cells primed by a mild pre-shock for 15 min at 48 °C before their exposure to the severe heat shock at 53 °C (48/53 °C) (Fig 1A). We experimentally established that 55 °C was an appropriate temperature to examine the impact of severe heat on *B*. *subtilis* cells growing on agar plates. In addition to exposure to these various heat conditions, we also examined other potentially proteotoxic stresses, such as salt and oxidative stress [9,17,48].

### Cellular (p)ppGpp levels increase during heat shock exposure

To investigate the impact of heat on the SR, we first assessed the intracellular levels of the alarmones pGpp, ppGpp and pppGpp ((p)ppGpp) during the heat shock response at 50 °C. We consider the sum of the cellular concentration of these three alarmones as a measure of the total (p)ppGpp alarmones concentration. Cells were grown at 37 °C in minimal medium to an optical density at 600 nm (OD_600nm_) of 0.4, and subsequently treated with a single, non-lethal temperature upshift to 50 °C in order to induce the heat shock response. After 2, 5 and 10 minutes of incubation at 50 °C, the intracellular levels of the alarmones were examined by liquid chromatography coupled mass spectrometry (LC-MS) (see Methods) (Fig 1) [49].

Already after 2 minutes, the alarmone levels increased approx. seven-fold (from 13 to 88 pmol OD^-1^ ml^-1^) (Fig 1B), which is in a similar range to that observed upon amino acid starvation induced by DL-norvaline (NV) (Fig 1C). In addition, significantly increased (p)ppGpp levels could be observed upon treatment with serine hydroxamate (SHX), salt stress induced by 6% (w/v) NaCl or 0.5 mM diamide, a strong oxidant of thiol groups (Fig 1C) [50,51]. It should be noted that (p)ppGpp levels increased only transiently during heat shock and returned to almost basal levels after 10 minutes (Fig 1B). Thus, we conclude that exposure to a non-lethal heat shock at 50 °C elicits a fast, but transient, increase of the (p)ppGpp alarmone levels.

We also assessed the levels of the alarmones under thermoresistance conditions (37/53 °C) and after priming (48 °C) under thermotolerance conditions (48/53 °C) (Fig 1A and 1D). We observed transiently increased (p)ppGpp levels (Fig 1D), however, the alarmone levels were particularly high during the severe heat shock shift at 37/53 °C (about 25-fold increase) and the induction was lower both for the 37/48 °C or 48/53 °C conditions (about 2–3 fold increase) (Fig 1D). The priming at 48°C appear to limit the alarmone synthesis of thermotolerant cells, when subsequently exposed to the lethal heat shock at 53 °C (Fig 1D).

The synthesis of (p)ppGpp that occurs during activation of the SR e.g. by treatment with serine hydroxamate or DL-norvaline, is in *B*. *subtilis* accompanied by a rapid decrease in the cellular GTP level [33], which we also observed after exposure to salt or diamide (S1A Fig). Interestingly, we did not observe a reduction in the GTP level after exposure to 50 °C (S1A Fig). The GTP level was at a relatively high level (S1B Fig) during temperature upshifts of 37/48 °C, 37/53 °C and 48/53 °C. However, after 15 min exposure to the raised temperature, the GTP level decreased in all temperature upshifts (S1B Fig).

Taken together, we observed that exposure to heat shock elicits a fast, but only transient, increase of the (p)ppGpp alarmones, which did not immediately affect the cellular GTP levels, which control the transcriptional response [33]. Therefore, it seems that alarmone levels might exhibit a graded response to heat exposure and temperature levels.

### Rel activity is the main source for (p)ppGpp synthesis during stress response

**Fig 2:**
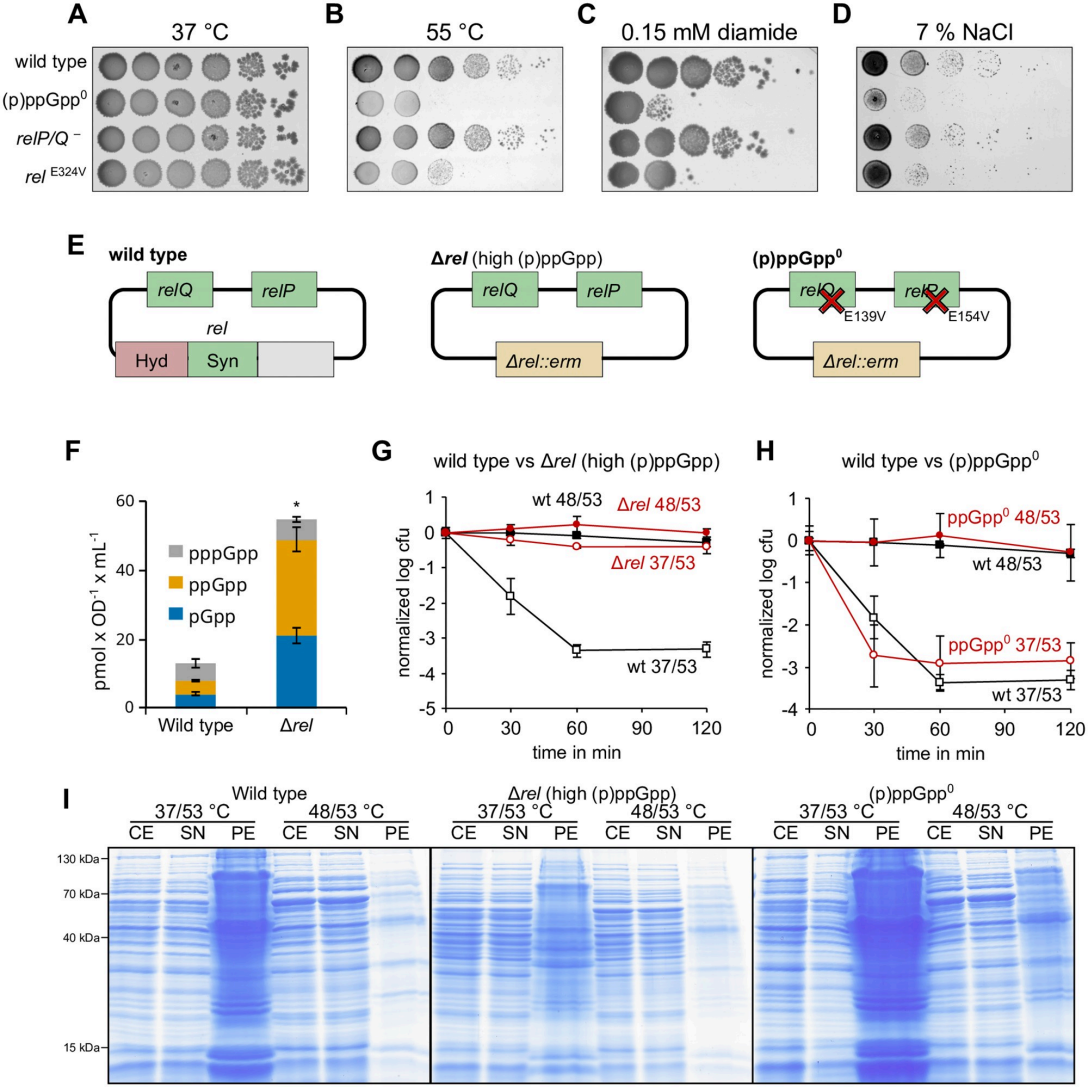
Increased (p)ppGpp levels confer high heat stress resistance. **(A-D)** Growth of strains with mutations in (p)ppGpp synthetases (*relP/Q*^-^: BHS204, *rel*^E324V^: BHS709; (p)ppGpp°: BHS214) on agar plates at 37 °C, during heat stress (55 °C), oxidative stress (0.1 mM diamide) or salt stress (total concentration of 7% (w/v) NaCl) over night. **(E)** Outline of the genotypes and the (p)ppGpp synthesis capabilities of the assessed wild type, Δ*rel* (BHS126 and BHS368) and (p)ppGpp° (BHS214 and BHS319) strains. **(F)** Cellular alarmone levels of wild type and Δ*rel* strain. Asterisks indicate significant changes (*p* ≤ 0.05) of combined pGpp, ppGpp and pppGpp levels according to Welch’s *t*-test. Means and SEM of three independent experiments are shown. **(G/H)** Thermotolerance and survival of wild type (black lines) and mutant strain (red lines) at 53 °C. Means and SEM of at least three independent experiments are shown. Open symbols: no pre-shock, closed symbols: 15 min pre-shock at 48 °C. (I) Accumulation of protein aggregates during heat stress at 53 °C without (37/53 °C) or with (48/53 °C) pre-shock. Exponentially growing cells of the indicated strains were shifted to 48 °C or left untreated for 15 min, then shifted to 53 °C for another 15 min. CE: cell extract, SN: supernatant, PE: pellet (aggregated protein fraction).

Next, we aimed to identify the source of (p)ppGpp during the heat stress response. To this end, strains with mutations that disrupt the (p)ppGpp synthetase activity of the proteins encoded by *relP* and *relQ* (*relP/Q*^-^ strain) or *rel* (*rel*^E324V^; inactive synthetase) were assayed for (p)ppGpp accumulation and GTP levels upon heat shock at 50 °C for 2 min (Fig 1E and S1C Fig). As a control, the (p)ppGpp accumulation was also measured in a (p)ppGpp° strain bearing inactivating mutations in all three alarmone synthetase genes (*relP*^*E154V*^, *relQ*^*E139V*^ and Δ*rel*) (Figs 1E and 2E). In addition, the (p)ppGpp-dependent transcription of *hpf* was employed as an additional read-out for the activation of the SR (S1D Fig) [52,53]. As expected, alarmone nucleotides were not detected in the (p)ppGpp° mutant under any conditions [28] (Fig 1E). We observed that upon heat exposure, the *relP*/*Q*^-^ (*relP*^*E154V*^, *relQ*^*E139V*^) strain also exhibited accumulation of (p)ppGpp (Fig 1E) and up-regulation of the *hpf* transcript similar to wild type cells (S1D Fig), indicating that the activity of RelP and RelQ is dispensable for (p)ppGpp production during heat stress. By contrast, the *rel*^E324V^ strain accumulated only small amounts of (p)ppGpp, with even lower levels after a brief heat exposure to heat (Fig 1E). Consistently, up-regulation of the *hpf* transcript in response to stress was also impaired in the *rel*^E324V^ strain (S1D Fig). Together, these results strongly suggest that the activity of Rel is the main source of (p)ppGpp during heat stress.

Activation of Rel during amino acid starvation requires the presence of uncharged tRNA and its association with the ribosome [21,22]. Early experiments by Cashel demonstrated that (p)ppGpp accumulation upon starvation for amino acids was almost completely suppressed in the presence of the translation inhibitor chloramphenicol, which indicated a connection between Rel activation and translation [54]. Interestingly, we also observed the same suppression of alarmone accumulation also upon heat and diamide treatment (Fig 1F). These experiments indicate that heat and oxidative stress mediated signal to activate Rel synthetase activity could be similar to the tRNA mediated signal activating the ribosome associated Rel upon amino acid starvation [19,20,54].

### *B*. *subtilis* cells lacking (p)ppGpp are more sensitive to stress

To assess the importance of alarmone production for cellular survival under heat stress, we monitored growth of wild type, (p)ppGpp°, *relP/Q*^-^ (*relP*^*E154V*^, *relQ*^*E139V*^) and *rel*^E324V^ *B*. *subtilis* strains at 37 °C and 55 °C on agar plates (Fig 2A and 2B). As expected, no obvious growth defect was observed for any of the strains at 37 °C. While the survival of the cells from the *relP/Q*—strain at 55 °C was identical to that of the wild type strain, strong growth defects were evident for the cells of the (p)ppGpp° and *rel*^E324V^ strains at 55 °C. These findings suggested that production of (p)ppGpp by Rel, but not RelP/Q, is critical for survival of *B*. *subtilis* cells under heat stress. Such severe growth defects were observed for both the (p)ppGpp° and *rel*^E324V^ strains not only under heat-, but also under oxidative- and salt stress, whereas the growth behavior of the *relP/Q*^-^ strain again resembled the wild type strain under the same conditions (Fig 2C and 2D). Collectively, these findings suggest that production of (p)ppGpp by Rel is critical for survival of *B*. *subtilis* cells, not only under heat stress, but also conditions of oxidative and salt stress.

### High cellular (p)ppGpp levels confer elevated heat stress resistance

Next, we asked whether (p)ppGpp levels influence thermotolerance development and survival. To do this, we utilized the (p)ppGpp° strain, which cannot synthesize (p)ppGpp (Fig 1E) as well as a Δ*rel* strain that exhibits constantly raised (p)ppGpp (Fig 2F) with concomitantly lowered GTP levels (S2A Fig). Rel is the only alarmone hydrolase in *B*. *subtilis* and the increased high (p)ppGpp levels in cells of *B*. *subtilis* Δ*rel* strain cause also an overall decrease in the growth rate (Fig 2F, S2A and S2B Fig), consistent with previous reports [28,29].

In the thermoresistance (37/53 °C) and thermotolerance (48/53 °C) experiments we observed that, unlike the cells of wild type, (p)ppGpp° or the Δ*relP* and Δ*relQ* cells strains (Fig 2G and 2H, S2C and S2D Fig), the Δ*rel* strain exhibited strongly increased thermoresistance, which was apparent from the high number of Δ*rel* cells still able to form colonies after the otherwise lethal heat shock (Fig 2G). Consistently, we also observed a strong reduction in protein aggregation during the 37/53 °C heat shock for the Δ*rel* strain, while the (p)ppGpp° strain exhibited more protein aggregation when exposed to the 37/53 °C heat shock (Fig 2I).

To investigate whether the increased heat resistance of the Δ*rel* strain was caused by the elevated levels of the alarmone (p)ppGpp, rather than the absence of the Rel protein, we expressed a truncated form of the *E*. *coli* RelA (*RelA*_*hyper*_) that exhibits constitutive and hyperactive alarmone synthetase activity *in trans* in wild type *B*. *subtilis* cells [55,56]. As a control, we also expressed a truncated form of the *E*. *coli* RelA (*RelA*_*inactive*_) that has no alarmone synthetase activity [55,56]. In a second approach, we examined *B*. *subtilis* Rel variants inactive in either the synthetase (Rel^E324V^) or hydrolase (Rel^H77A/D78A^) expressed *in trans* in the *B*. *subtilis* (p)ppGpp° strain.

Expression of *E*. *coli* RelA_hyper_ or hydrolase-inactive *B*. *subtilis* Rel^H77A/D78A^ *in trans* resulted in increased alarmone levels (S3A Fig) and conferred high thermoresistance (S3B and S3C Fig), as observed for the *B*. *subtilis* Δ*rel* strain (Fig 2G). By contrast, *B*. *subtilis* (p)ppGpp° strains expressing *E*. *coli* RelA_inactive_ or the *B*. *subtilis* synthetase-inactive Rel^E324V^ *in trans* displayed neither increased alarmone levels (S3A Fig), nor increased survival to severe heat stress (S3D and S3E Fig). This suggests that the increased alarmone levels, independent of the synthetase, are responsible for the thermoresistance phenotype in *B*. *subtilis*.

### The role of cellular GTP levels during heat stress

High (p)ppGpp levels during the SR lead in *B*. *subtilis* to a decrease in the cellular GTP level and this decrease is known to be intricately involved in causing the transcriptional changes during the SR [33,34] (S1, S2A and S3A Figs). To examine, whether the resistance to heat stress observed in the Δ*rel* strain could be mediated simply by lowering the cellular GTP level, wild type cells were treated with decoyinine, an inhibitor of GMP synthetase, which decreases the cellular GTP level (> 3-fold) without increasing (p)ppGpp levels [57,58]. Treatment with 50 μg ml^-1^ decoyinine showed no effect, while addition of 250 μg/ml decoyinine resulted in a partially increased thermoresistance. (S4 Fig). However, further increased decoyinine concentrations reduced (400 μg/ml), or even abolished (1000 μg/ml) both thermoresistance and thermotolerance development (S4 Fig). These experiments suggest that the decoyinine-mediated lowered cellular GTP level, which is a prerequisite for the reprogramming of the transcriptome during SR [33,34,59], can elicit heat resistance only to a limited extent. However, the observed effect of decoyinine on thermoresistance was weaker in comparison to the effect of raised (p)ppGpp levels (Fig 2F and 2G and S3 Fig).

From these observations, we infer that raised (p)ppGpp levels are sufficient to confer increased stress resistance and reduced levels of heat-induced protein aggregates. However, the SR-mediated drop in the cellular GTP level [33] was not observed during the heat shock response (S1 Fig) and an artificial reduction of the cellular GTP level by decoyinine had only a moderate effect on thermoresistance and could even abolish thermotolerance (S4 Fig).

Therefore, we went on and investigated the transcriptome, translation and proteome at raised temperatures and in the absence and presence of (p)ppGpp.

### Transcriptome changes in the presence and absence of (p)ppGpp at raised temperatures

We performed global RNA-seq experiments to compare the transcriptome changes between *B*. *subtilis* wild type, Δ*rel* and (p)ppGpp° strains under exponential growth (37 °C) after heat shock (15 min 48 °C) as well as the thermotolerance conditions 37/53 °C and 48/53 °C (Fig 1A) in wild type cells. Since down-regulation of “stable” rRNA is a hallmark of the SR, we introduced a previously established chromosomal *rrnJ*p1-*lacZ* fusion into the tested strains, which allowed us to follow the activity of this rRNA promoter with RNA-seq and RT-qPCR experiments in these *B*. *subtilis* strains [17].

**Fig 3:**
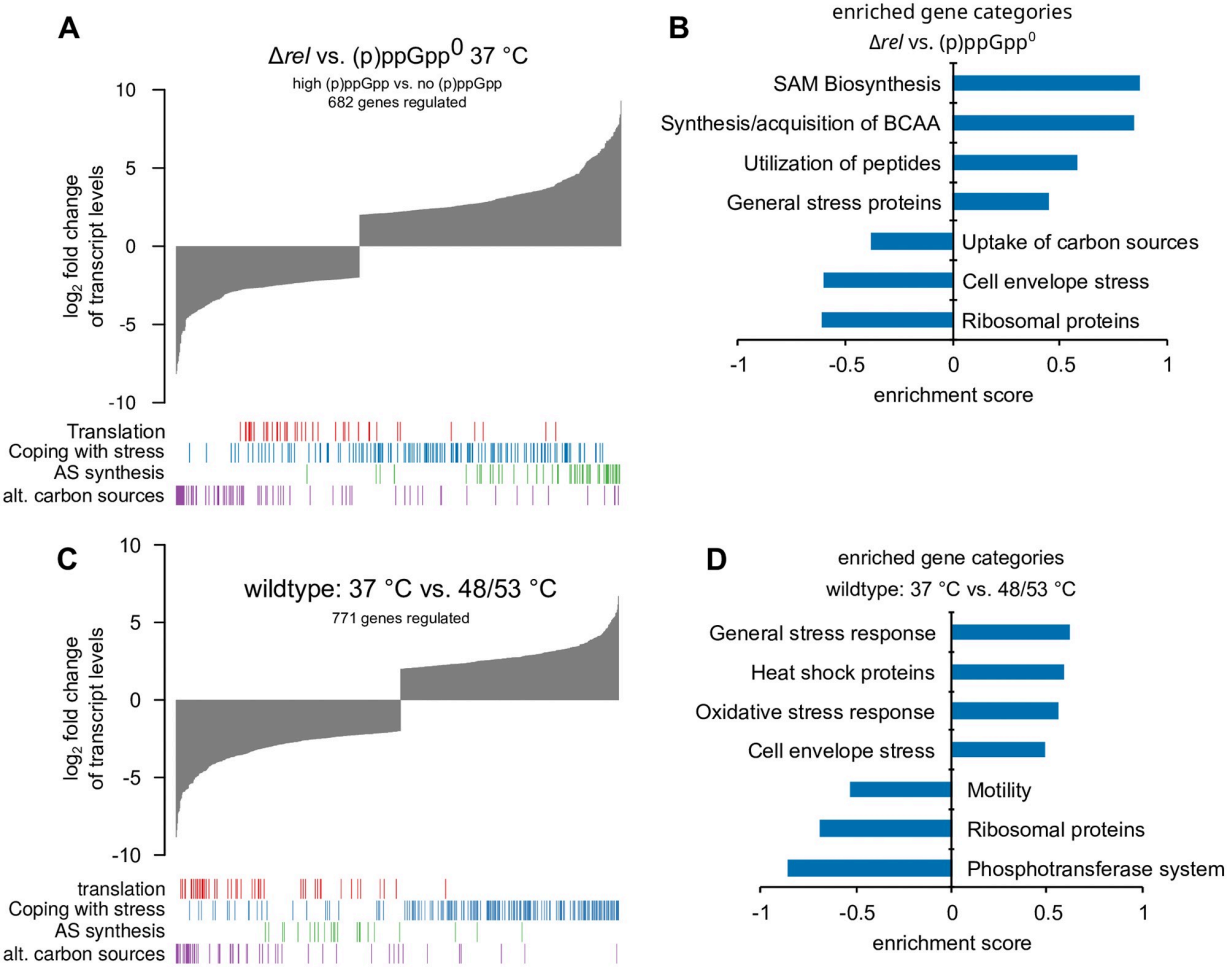
(p)ppGpp- mediated global changes in the transcriptome. **(A)** Global differences in gene expression in Δ*rel* versus (p)ppGpp° strains. Bar tracks indicate the distribution of genes in the respective functional groups. **(B)** Selected category results of the gene set enrichment analysis from regulated transcripts in Δ*rel* vs. (p)ppGpp° cells. Positive/negative enrichment scores represent enrichment in the up- or down-regulated genes. **(C)** Global differences in gene expression in exponentially growing (37 °C) or thermotolerant (48/53 °C) wild type cells. Bar tracks indicate the distribution of genes in the respective functional groups. **(D)** Selected category results of the gene set enrichment analysis from regulated transcripts in unstressed (37 °C) or thermotolerant (48/53 °C) wild type cells. Positive/negative enrichment scores represent enrichment in the up- or down-regulated genes.

**Fig 4:**
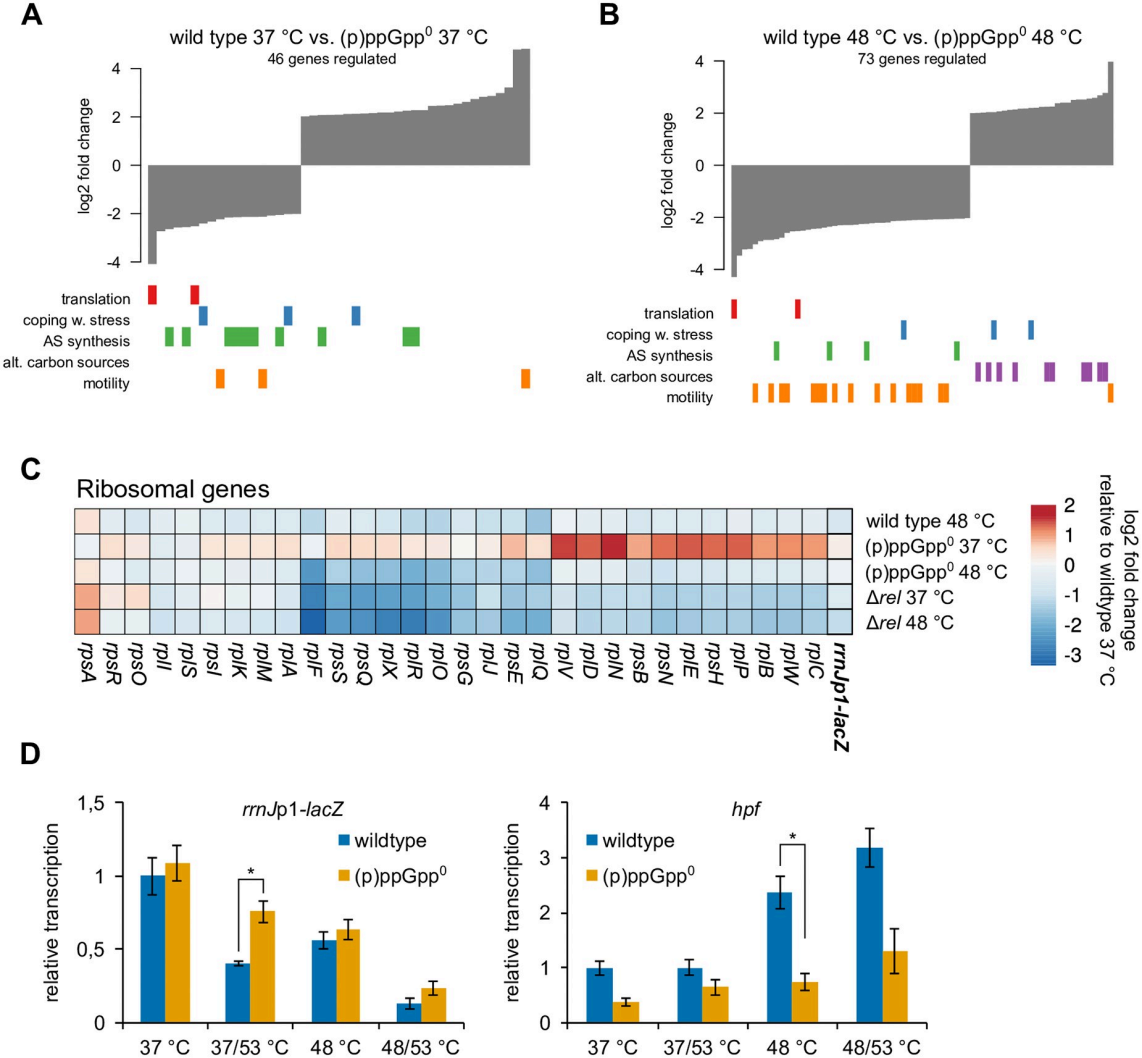
(p)ppGpp mediated transcriptional changes during heat stress. **(A/B)** Global differences in gene expression in wild type versus (p)ppGpp° strains at 37 °C or 48 °C, respectively. Bar tracks indicate the distribution of genes in the respective functional groups. **(C)** Heatmap showing expression changes of selected transcripts during mild heat stress in wild type, (p)ppGpp° or Δ*rel* cells. Values represent log_2_ fold changes of transcript levels relative to wild type cells at 37 °C. **(D)** Relative changes in the transcription of selected genes during heat shock in wild type and (p)ppGpp° strains determined by RT-qPCR. Means and SEM of three replicates are shown. Asterisks indicate significance (*p* ≤ 0.05) according to Welch’s *t*-test.

Thereby we were able to gain insights into (i) the strong phenotype associated with a *rel* deletion especially when compared with the (p)ppGpp° *B*. *subtilis* strain (Fig 3A and 3B) and compare it to the (ii) transcriptome changes of wild type cells during thermotolerance 37 vs 48/53 °C (Fig 3C and 3D). At the same time (iii) the transcription pattern of all gene sets of interest could be compared and inspected for all the tested conditions and introduced mutations (Fig 4, S5, S6 and S7 Figs). In addition, we used RT-qPCR experiments to validate our RNA-seq experiment and to investigate different conditions such as growth at 50 °C.

#### Stringent response

First, we analyzed the transcriptomic data from the exponentially growing wild type, (p)ppGpp° and Δ*rel* strains (Fig 3A and 3B, S1, S2 and S3 Datasets). By comparing Δ*rel* cells, which exhibit constitutively high alarmone levels (Fig 2F), with (p)ppGpp° cells, 682 genes were found to be regulated by (p)ppGpp (Fig 3A) and we observed a good correlation with RT-qPCR experiments of selected regulated genes (S5A Fig). We noticed a broad down-regulation of many translation-related genes including the *rrnJ*p1*-lacZ* reporter as well as an extensive de-repression of CodY-controlled amino acid synthesis genes (e.g. *ilvB* (Fig 3A and 3B, S5B and S6 Figs)), both of which are characteristic for the SR in accordance with previous transcriptomic studies [37,53,60,61]. Furthermore, a strong decrease in the transcription of CcpA-regulated genes required for the utilization of alternative carbon sources was observed (e.g. *rbsC* (Fig 3A and 3B, S5B and S6 Figs)). Interestingly, the transcription of many heat shock genes was decreased in Δ*rel* cells (e.g. *dnaK* and *clpE* (S5B Fig)). In contrast, we noticed increased transcript levels of many general stress genes of the SigB regulon (e.g. *ssrA*, *dps*, *gsiB*, *ysnF*) in the absence of stress at 37 °C Δ*rel* cells (Fig 3B and S5B Fig). Notably, the transcript level of *hpf* (*yvyD*), encoding the hibernation promoting factor Hpf, was increased by raised (p)ppGpp levels (24-fold up-regulated, (S5A and S5B Fig)), confirming that the increased transcription of *hpf* can be considered as a reporter for the activation of the SR [52,53].

#### The heat-induced (p)ppGpp pulse mediates only minor transcriptional changes

After having established that alarmones can play a protective role during the heat shock response, we sought to assess the role of (p)ppGpp in transcriptional changes during heat exposure. To this end, we analyzed the thermoresistance (37/53 °C) and thermotolerance (48°/53 °C) conditions (Figs 1A, 3C and 3D) [9,17] in wild type cells and investigated the wild type, (p)ppGpp° and Δ*rel* strains also at 48 °C, of the same RNA-seq experiment. Overall, we detected only small changes when comparing the transcriptome of wild type cells and (p)ppGpp° cells at 37 °C and 48 °C, indicating that the majority of transcriptional changes of the heat stress response are mediated independently of (p)ppGpp (Fig 4A and 4B). Thermotolerant wild type cells (48/53 °C) exhibited a comprehensive down-regulation of translation-related genes including the *rrnJ*p1-*lacZ* reporter that was, to a lesser extent, also observed in the mild pre-shock (48 °C) and severe heat shock (37/53 °C) conditions (Figs 3 and 4, S5 and S6 Figs), in agreement with previous observations [17]. Importantly, the heat-mediated down-regulation of *rrnJ*p1-*lacZ* appeared to be partially (p)ppGpp-dependent and was therefore less pronounced in the (p)ppGpp° strain (Fig 4C and 4D). Independent RT-qPCR experiments confirmed this observation. The requirement of (p)ppGpp for repression of *rrnJ*p1 under heat stress became even more apparent when the 50 °C heat shock condition was examined (S7A Fig).

By contrast, the heat-mediated down-regulation of many ribosomal protein genes and other translation-related genes appeared to also occur in the absence of (p)ppGpp, indicating a more complex and (p)ppGpp-independent control of the transcription of these genes (Fig 4C). Furthermore, while an extensive de-repression of the CodY regulon could be observed in Δ*rel* cells as a hallmark of the SR, no increased transcription of CodY-regulated genes was observed in any of the heat shock conditions tested (S6 and S7B Figs), which could be explained by the unchanged GTP level in heat shocked cells (S1 Fig).

The transcript level of the genes encoding conserved chaperones and proteases of the heat shock response regulon were strongly up-regulated upon all temperature up-shifts, independently of the presence or absence of (p)ppGpp (S6 and S7C Figs). Interestingly, additional RT-qPCR experiments performed with RNA from 50 °C heat shock-treated cells revealed that the heat-induced expression of some SigB-regulated genes was impaired in the (p)ppGpp° background, e.g. *ssrA* (approx. 2-fold lower expression in (p)ppGpp° cells at 50 °C) and *dps* (approx. 3-fold lower expression), indicating a possible functional connection between the SR and the general stress response (S7C and S7D Fig) [62,63]. However, the majority of genes of the SigB regulon were found to be induced in the (p)ppGpp° strain similarly to wild type cells at 48 °C (S6 Fig).

Notably, the heat-induced expression of *hpf*, which is positively regulated by the SR (S5A Fig), was lower in the (p)ppGpp° strain compared to wild type cells during heat stress (Fig 4D and S7A Fig). Furthermore, while CcpA-regulated genes were repressed in wild type and (p)ppGpp° cells under heat shock conditions (Fig 3 and S6 Fig), some genes (e.g. *rbsD*, *ganP*, *licH*) were less down-regulated or even induced at 48 °C in the (p)ppGpp° strain (Fig 4B). In contrast, motility-genes were particularly strongly down-regulated by heat in the (p)ppGpp° mutant (Fig 4B, S5 and S6 Figs), while the down-regulation of these genes appeared not to be significant in wild type cells at 48 °C (median 1.14-fold change, S6 Fig) [64].

Taken together, (p)ppGpp has a small but noticeable impact on the transcriptome during heat stress. The heat-mediated up-regulation of *hpf* and the down-regulation of *rrnJp1-lacZ* appear to be dependent on (p)ppGpp. However, the overall induction of the heat shock response as well as the strong repression of many ribosomal protein genes observed during heat stress appear to be mostly independent of alarmones. The induction of the CodY regulon, a hallmark of SR, was also not much effected during heat stress, most likely because the heat mediated transient increase of (p)ppGpp might not be sufficient to lower the cellular GTP level for the subsequent remodeling of the transcriptome known from the fully induced SR [60].

It should be noted that when designing the RNA-seq experiment, we choose 48 °C as a simple heat shock condition for the mutant strains since it resembled the thermotolerance protocol (Fig 1A) and the condition of previously published microarrays [17]. However, many phenotypes of Spx and (p)ppGpp could be observed best upon a stronger, but non-lethal, heat shock at 50 °C [17], which we could assess by RT-qPCR. We also observed that, while wildtype cells treated with 37/53 °C exhibit a strong increase of (p)ppGpp within the first minutes of stress (Fig 1D), the examination of cellular physiology under these lethal conditions might be influenced by the apparent reduction of viability of about one order of magnitude (Fig 2G) [9,17].

### Complementary roles of Spx and stringent response during heat shock

**Fig 5:**
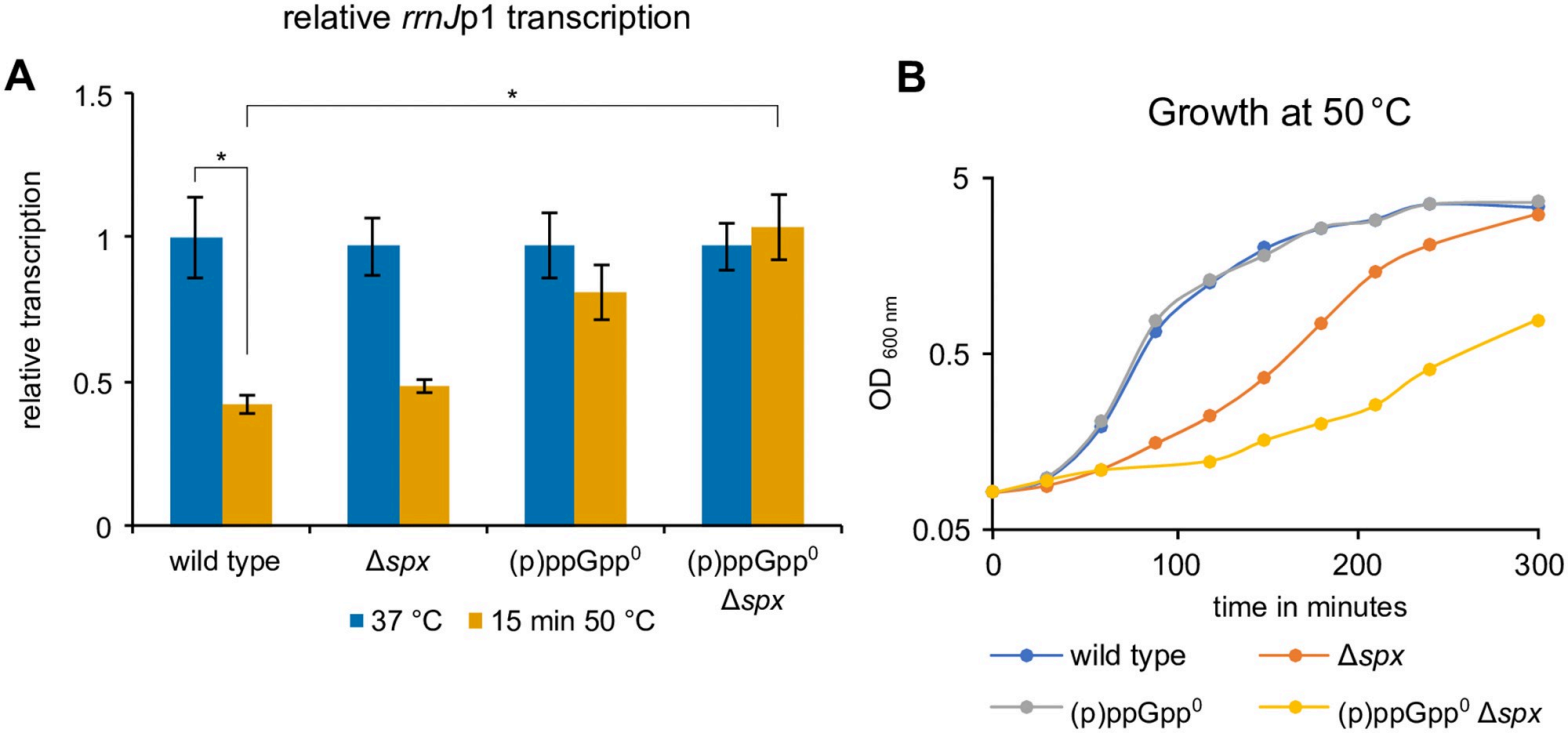
Complementary roles of (p)ppGpp and Spx under heat stress. **(A)** Heat mediated down-regulation *of rrnJ*p1-*lacZ* transcription in wild type (BHS220), Δ*spx* (BHS222), (p)ppGpp° (BHS319) and Δ*spx* (p)ppGpp° (BHS766) strains as determined by RT-qPCR. Means and SEM of three independent experiments are shown. Asterisks indicate significance (*p* ≤ 0.05) according to Welch’s *t*-test. **(B)** Growth of the same strains in LB medium at 50 °C.

Previously, we reported that Spx, a central regulator of the heat- and oxidative stress response, can down-regulate the transcription of translation-related genes and rRNA [17]. However, an *spx* deletion strain was not impaired in the heat-mediated down-regulation of these genes [17]. Here, we noticed a detectable, albeit limited, involvement of the SR in the transcriptional down-regulation of specific genes during heat stress (*rrnJ*p1*-lacZ*), suggesting an intricate regulation of these genes by different factors. To test for such a concurrent and complementary transcriptional regulation by Spx and (p)ppGpp, a *B*. *subtilis* strain combining a *spx* deletion with the (p)ppGpp° mutations was constructed. Down-regulation of *rrnJ*p1-*lacZ* upon heat shock appear to largely depend on (p)ppGpp (Fig 5A), however Spx can also repress this promoter also in the absence of (p)ppGpp (S9B Fig) [17]. Interestingly, this (p)ppGpp° Δ*spx* strain also displayed a slow growth phenotype at 37 °C and a more severe growth defect at 50 °C compared to the strains with single deletions of (p)ppGpp° or Δ*spx* (Fig 5B and S8B Fig). These findings suggest a possible genetic interaction of the SR and the *spx* regulon under heat stress conditions. Consistently, the (p)ppGpp° Δ*spx* strain accumulated more heat-induced protein aggregates at 50 °C than cells lacking either (p)ppGpp or *spx* (S8C Fig). However, the transcription of selected r-protein genes was also down-regulated in the (p)ppGpp° Δ*spx* strain (S8A Fig), suggesting additional factors beyond Spx and (p)ppGpp, that can also influence the promoter and/or the stability of these transcripts.

When mutations in *rpoA* were introduced in the (p)ppGpp° strain that abolish Spx-mediated up- and down-regulation (*cxs*-1/*rpoA*^Y263C^), or interfere only with Spx-mediated repression of rRNA while still allowing up-regulation of redox chaperones (*cxs*-2 / *rpoA*^V26^°^A^) [17], only the (p)ppGpp° *cxs*-1 strain displayed a severe growth defect as observed for the (p)ppGpp° Δ*spx* strain (S8B Fig). This experiment suggests that the Spx-mediated up-regulation of stress response genes, and not the ability to down-regulate translation-related genes, is required for efficient growth in the (p)ppGpp° background. Notably, (p)ppGpp is sufficient for the down-regulation of translation-related genes during norvaline-induced amino acid limitation, while Spx is dispensable for this process (S9A Fig). Conversely, Spx can act on rRNA promoters independently of (p)ppGpp *in vivo* (S9B Fig) [17]. In addition, *in vitro* transcription experiments with purified Spx and RNAP gave no indications that ppGpp could directly influence Spx mediated transcriptional activation or inhibition of RNAP (S9C Fig). Furthermore, Spx-dependent stress response genes (e.g. *trxB*, *clpX*) are not up-regulated in the Δ*rel* strain (S3 Dataset), suggesting that Spx is not activated by (p)ppGpp *in vivo*.

Together, these experiments suggest a complex interplay between Spx and (p)ppGpp during the heat shock response and that the activity of at least either Spx or (p)ppGpp is important for efficient growth during heat stress. However, the inhibitory activity of Spx on translation-related genes appears to be dispensable for stress tolerance and many r-protein genes were down-regulated during heat stress even in the absence of both Spx and (p)ppGpp.

The observation that transcription of *spx* is also activated by (p)ppGpp via CodY in *Enterococcus faecalis* and that *rel* transcription is activated by the disulfide-stress regulator σ^R^ in *Streptomyces coelicolor* points toward a possible functional connection of these two regulators [65,66].

### (p)ppGpp regulates translation during heat stress

Upon heat shock, we observed raised levels of (p)ppGpp, but not the transcriptional reprogramming triggered by lowered GTP levels (Figs 3 and 4, S6 and S7 Figs). Therefore, we wanted to determine the impact of (p)ppGpp on translation during heat stress. To this end, a method for pulse-labeling newly synthesized nascent peptide chains using puromycin was utilized to estimate protein synthesis rates (see Methods, S10 Fig) [67]. As expected, expressing the small alarmone synthetase *relP* (*ywaC) in trans*, results in accumulation of high (p)ppGpp levels, which concurrently lead to a strong decrease in translation rate, indicating that translation is inhibited in these cells (S10D and S10E Fig) [39,53,68].

**Fig 6:**
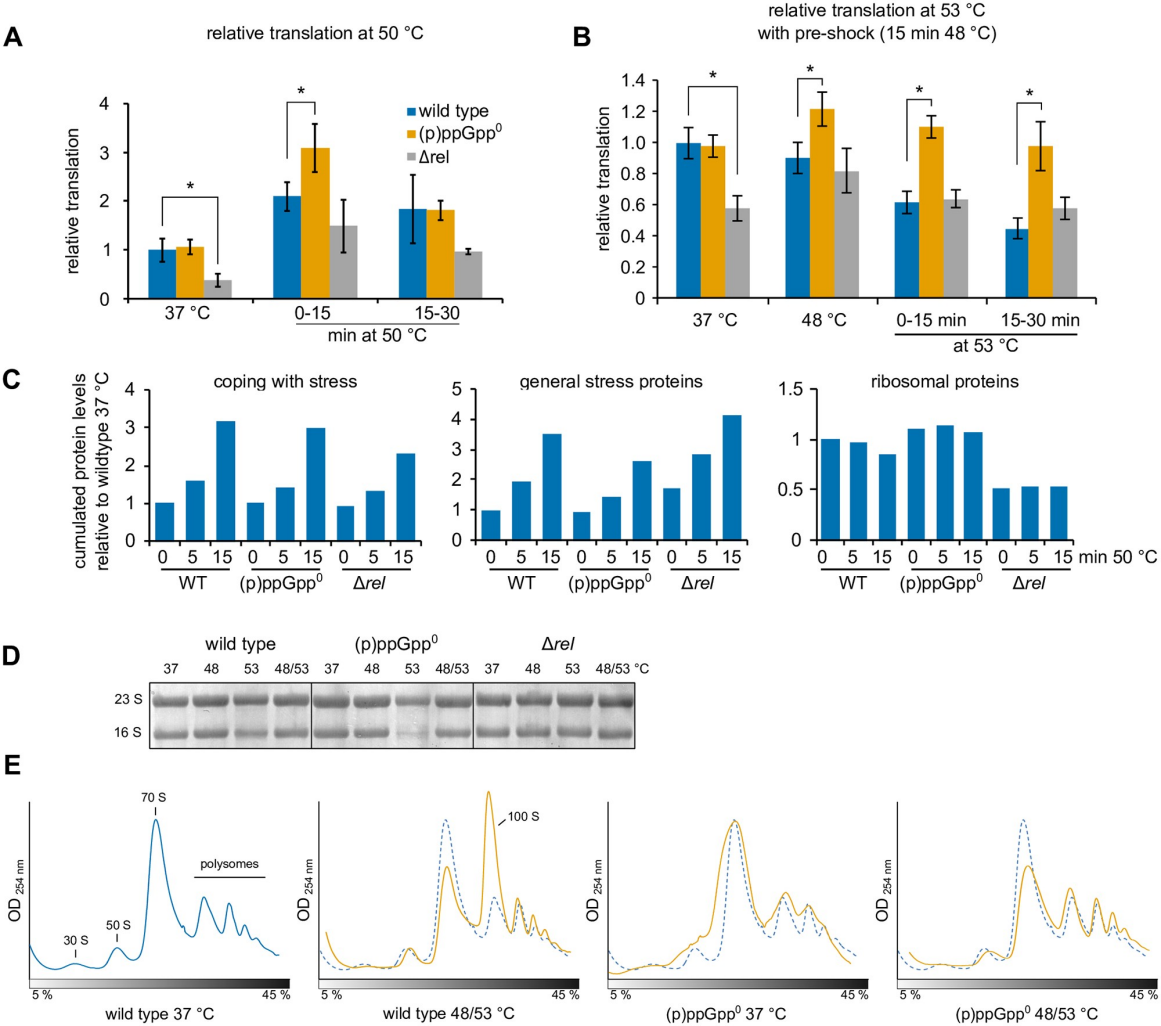
(p)ppGpp modulates translation during stress response. **(A/ B)** Relative translation (estimated from puromycin incorporation) of wild type, (p)ppGpp° (BHS214) and Δ*rel* (BHS126) strains during heat stress **(A)** at 50 °C or **(B)** at 48 °C or 48/53 °C. 1 μg ml^-1^ puromycin was added for 15 min to the medium directly after (0–15 min) or 15 min after shifting the sample to the indicated temperatures. Means and SEM of four independent experiments are shown. Asterisks indicate significance (*p* ≤ 0.05) relative to wild type according to Welch’s *t*-test. **(C)** Relative cumulated protein levels of selected categories of wild type, (p)ppGpp0 and Δ*rel* strains during heat stress. Categories were inferred from SubtiWiki. The relative abundances of all proteins of the respective category were cumulated and normalized to the control condition (wild type 37 °C). **(D)** Methylene blue stained membranes showing the integrity or degradation of rRNA after severe heat stress (53 °C). Wild type, (p)ppGpp° (BHS214) or Δ*rel* (BHS126) cells were heat-shocked at 48 °C, 53 °C or 48/53 °C for 15 min each. 2 μg total RNA was separated on denaturing agarose gels and blotted on nylon membranes. **(E)** Sucrose gradient profiles of extracts from untreated (37 °C) or thermotolerant (48/53 °C for 15 min each) wild type or (p)ppGpp° (BHS214) cells. The dashed blue line of untreated wild type cells is shown for reference.

When we examined the translation rate in cells, we observed that the Δ*rel* strain always exhibited a lower translation rate compared to wild type cells at 37°C (Fig 6A and 6B), consistent with its raised (p)ppGpp levels and the observed “stringent” phenotype of this strain. The difference between wild type and Δ*rel* strain diminished upon exposure to higher temperatures, possibly influenced also by stress induced raised levels of the alarmone in wild type cells at these higher temperatures. By contrast, the “relaxed” (p)ppGpp° strain always exhibited higher translation rates (Fig 6A and 6B), indicating a more deregulated translation compared to wild type or Δ*rel* strains. During the non-lethal 50 °C heat shock, translation rates transiently increased in all strains (Fig 6A). Nevertheless, the (p)ppGpp° strain still displayed significantly higher translation rates compared to wild type and the Δ*rel B*. *subtilis* strains (Fig 6A and 6B). The observed influence of (p)ppGpp on translation suggests that the most important impact of (p)ppGpp under heat stress appears not to be its effect on transcription (Fig 4 and S5 Fig), but the direct modulation of translation (Fig 6), possibly by directly interfering with the activity of different translational GTPases [39,40,68,69].

Down-regulation of translation accompanied by slower growth that increases thermoresistance can also be observed in cells with defective ribosomes, lacking e.g. the ribosomal protein L11 (RplK). RplK is not essential, however the Δ*rplK* strain exhibits severe translation defects and a strongly reduced growth rate (S11 Fig), despite its inability to synthesize (p)ppGpp [70]. Such a *B*. *subtilis* Δ*rplK* strain exhibits increased heat stress tolerance similar to Δ*rel* cells (S11 Fig). This observation suggests that a reduced translation rate caused by a defective ribosome is sufficient to increase survival under heat stress, even in the absence of the alarmone. In summary, these observations indicate that the intracellular (p)ppGpp second messenger can immediately modulate translation during heat stress and that the reduction of the protein synthesis rate *per se* can promote increased stress tolerance.

### Changes in protein levels mediated by heat shock and (p)ppGpp

The observation that (p)ppGpp appears to directly regulate translation under heat stress prompted us to also examine the effect of the alarmones and heat on changes in the proteome. Therefore, we employed mass spectrometry for a proteome-wide identification and quantification of cellular proteins from stressed (15 min 50 °C) and unstressed (37 °C) wild type, (p)ppGpp° and Δ*rel* cells. In total, we quantified 2641 proteins which were identified with at least two peptides in all conditions (S4 and S5 Datasets, S12 and S13 Figs). Under heat stress (50 °C), a pronounced increase of heat-specific stress response proteins, e.g. ClpC or GroEL, was observed in all strains, indicating that the translational capacity is sufficient to promote the synthesis of heat shock proteins in wild type, and even in the Δ*rel* strains where the translation rate is reduced (Fig 6C and S12 Fig).

In contrast, the heat mediated synthesis of SigB-controlled general stress proteins was reduced in (p)ppGpp° cells, whereas their levels were increased in the Δ*rel* strain, which corroborates the regulatory connection between alarmone synthesis and the general stress response already observed in the RNA-seq experiment (Fig 6C and S12 Fig).

Abundant ribosomal proteins represent a large proportion of the cellular protein mass in wild type and (p)ppGpp° cells, however their levels were strongly decreased in Δ*rel* cells in accordance with the constitutive stringent regulation observed in this strain (Fig 6C, S12 and S13 Figs). Interestingly, the levels of many r-proteins appear to be reduced upon heat stress in wild type but not in (p)ppGpp° cells (Fig 6C and S12 Fig), which supports an alarmone dependent post-translational control mechanism of the abundance of these translation-related proteins.

When comparing the proteomes of Δ*rel* cells with wild type cells under unstressed conditions, we observed large-scale changes that resembled in many ways the results obtained by RNA-seq (S13 Fig). In contrast, the differences in the proteome between wild type and (p)ppGpp° cells were comparatively smaller at both 37 °C and 50 °C (S13 Fig). Importantly, a gene set enrichment analysis of the differentially regulated proteins from wild type or (p)ppGpp° at 37 °C or 50 °C revealed only few enriched functional categories (S5 Dataset). These observations suggest, that (p)ppGpp is involved in the regulation of the total translation capacity.

In summary, although (p)ppGpp may be involved in post-transcriptional regulation of some proteins, it appears that the alarmones assist in the development of stress tolerance by controlling global changes in translation rate. However, we observed a (p)ppGpp-dependent regulation of specific protein classes of the proteome. (Fig 6C). Notably we observed a reduction of r- proteins depending on the alarmone and a relative increase in chaperone levels during heat shock independent of the alarmone (Fig 6C, S12 and S13 Figs). Since we observed the heat mediated induction of chaperones even in the *rel* strain with its constantly elevated (p)ppGpp levels and a slowed down translation, we suspect the possibility of a mechanism allowing the specific translation of chaperones, albeit the generally slowed down translation.

In addition to this protective function, (p)ppGpp, which is synthesized only as a pulse by Rel during relatively uninhibited growth at 50 °C, could modulate or inhibit translation, presumably by directly interfering with translation factors [38,39,41,69]. A set of interesting *in vitro* experiments suggested a modulating effect of ppGpp and (p)ppGpp on IF2 and translation initiation, which also depended on specific structured elements of translated mRNA, allowing translation of specific mRNA’s in the presence of alarmones [41]. These experiments indicate a specific ability of alarmones, interacting with IF2, to limit translation in general, while possibly still allowing the expression of specific genes necessary for stress resistance [41].

### (p)ppGpp is required for ribosome integrity and 100S formation during heat stress

Treatment with a lethal temperature shift (37/53 °C) without pre-shock resulted in a strong decrease in translation efficiency in wild type and (p)ppGpp° strains. Interestingly, translation was strongly decreased in (p)ppGpp° cells at 37/53 °C, while wild type cells still maintained active translation under these conditions (S14A Fig). The lowered translation activity in (p)ppGpp° cells appears to be accompanied by a strong reduction of the levels of cellular 16S rRNA during this severe heat shock (Fig 6D). This could indicate a defect in 16S rRNA maturation and the assembly and/or activity of the small ribosomal subunit, which would be consistent with the observed heat sensitivity of the *B*. *subtili*s (p)ppGpp° strain (Fig 2A and 2B). In contrast, the translation in Δ*rel* cells appeared to be transiently increased in comparison to the wild type and (p)ppGpp° strains (S14A Fig), in agreement with the observed high heat-resistance of this strain to the otherwise lethal heat shock, which negatively affects the growth of the more sensitive wild type and (p)ppGpp° strain (Fig 2G).

The (p)ppGpp° strain also failed to induce expression of the *hpf* gene during heat stress and did not accumulate the Hpf protein (Fig 4D and S14B Fig). Thus, the formation of 100S disomes upon heat stress, which was clearly visible in the ribosome profiles of wild type and Δ*rel* cells (especially under thermotolerance conditions) was abolished in the (p)ppGpp° strain where only polysomes could be detected similarly as in Δ*hpf* cells (Fig 6E and S14C Fig) [71]. However, the apparent decrease in the 16S rRNA observed under severe stress conditions was not prevented by *in trans* expression of Hpf (S14D Fig) and overexpression of Hpf could not rescue the heat-sensitive phenotype of (p)ppGpp° strains (S14E Fig). Also, the addition of translation-inhibiting antibiotics could not rescue this phenotype, indicating that inhibition of translation *per se* is not sufficient to protect ribosomes during severe heat stress (S14F Fig).

It was recently observed in *B*. *subtilis* that tRNA maturation defects could lead to an inhibition of rRNA processing and 30S assembly via the synthesis of (p)ppGpp [72], supporting a role of the alarmone in ribosome maturation.

Taken together, these observations indicate that (p)ppGpp is also required for the integrity of the ribosomal subunits and the formation of 100S particles under heat stress. These observations might be important to understand possible stress signaling pathways and also the protective effects of (p)ppGpp on translation under proteotoxic stress conditions.

### The activation of the stringent response during heat stress

The results presented here clearly reveal that (p)ppGpp accumulates rapidly during heat and other environmental stresses (Fig 1). In addition, strains unable to synthesize (p)ppGpp are rendered sensitive to high temperatures and accumulate more heat-induced protein aggregates (Fig 2A–2D, 2H and 2I). Interestingly, (p)ppGpp synthesis and heat tolerance are solely dependent on the synthetase activity of Rel, indicating that this enzyme is responsible for the synthesis of (p)ppGpp under these conditions (Figs 1E and 2A–2D).

Similarly to heat stress, disulfide or salt stress can also lead to inactivation, unfolding and aggregation of proteins [48,73]. It is possible that protein aggregation or inactivation could be involved in the signal for the stress-mediated activation of Rel, since proteotoxic and oxidative stress can result in the inactivation of enzymes and may impair uptake or biosynthesis of certain amino acids [51,74–76].

The transcriptional and translational heat shock response, which usually depends on sensing temperature indirectly or directly by cellular protein unfolding is in the range of 2–5 min [5,6]. Since the protein unfolding precedes the transcriptional or translational response, one can estimate that cellular protein unfolding upon sudden proteotoxic stress happen faster than 2–3 min. This is also consistent with the observation of the *in vivo* formation of subcellular protein aggregates, which are preceded by unfolding and misfolding events, can already be observed about 2 min after heat shock [7]. The relatively fast and transient kinetics of heat induced alarmone synthesis (Fig 1) would therefore be consistent with the time frame known from general heat mediated protein misfolding and the unfolding or misfolding of specific stress-sensor proteins during heat stress.

Our experiments demonstrate that Rel activation during heat- or oxidative stress can be inhibited by chloramphenicol, similarly as during amino acid starvation (Fig 1F and S1E Fig). Therefore, the underlying activation mechanisms during environmental stress likely share some similarities to the well-studied SR-activation upon amino acid deprivation and may also involve the sensing of uncharged tRNA on the ribosome [21,22,74,77].

The stress induced depletion of amino acids can result in the accumulation of uncharged tRNA, which serves as a signal to activate Rel. In addition, tRNAs and proteins of the translational machinery are prone to oxidation or modification upon stress, leading to translation stalling, which can also elicit the SR [78]. For example, oxidation of tRNAs at a conserved 4-thiouridine residue reduced the affinity for their cognate aminoacyl-tRNA synthetase, which was found to be the basis for the activation of the SR upon UV-exposure in *S*. *enterica* [79].

It is very likely that the heat stress signal is first sensed via unfolded proteins which might then be transmitted by specific tRNA to Rel on the ribosome by the various discussed possibilities. However, it cannot be excluded that e.g. Rel itself can act as a heat stress sensing protein on the ribosome, or that a heat stress sensing protein that interacts with Rel could be involved, as for example suggested for competence development in *B*. *subtilis* [80]. Clearly, more experiments are required to identify the molecular mechanism underlying the activation of Rel and the control of the SR during heat stress in *B*. *subtilis*.

### The role of (p)ppGpp and SigB upon heat stress

Both the transcriptomic and the proteomic datasets also indicate a possible activation of the SigB-dependent general stress response by (p)ppGpp during stress- and non-stress conditions (Figs 3B and 6C, S5B, S7, S12 and S13 Figs). SigB becomes activated by decreased GTP levels as elicited by decoyinine [62,63]. In addition, a requirement of L11, which is necessary for Rel synthetase activity, and Obg, a ribosome-associated GTPase that interacts with (p)ppGpp, for the activation of SigB upon physical stress and an interaction of Obg with components of the SigB regulatory cascade was reported, suggesting an intricate connection between the ribosome, Rel and the activation of the general stress response [62,70,81].

### The role of the SR during the heat stress response

**Fig 7:**
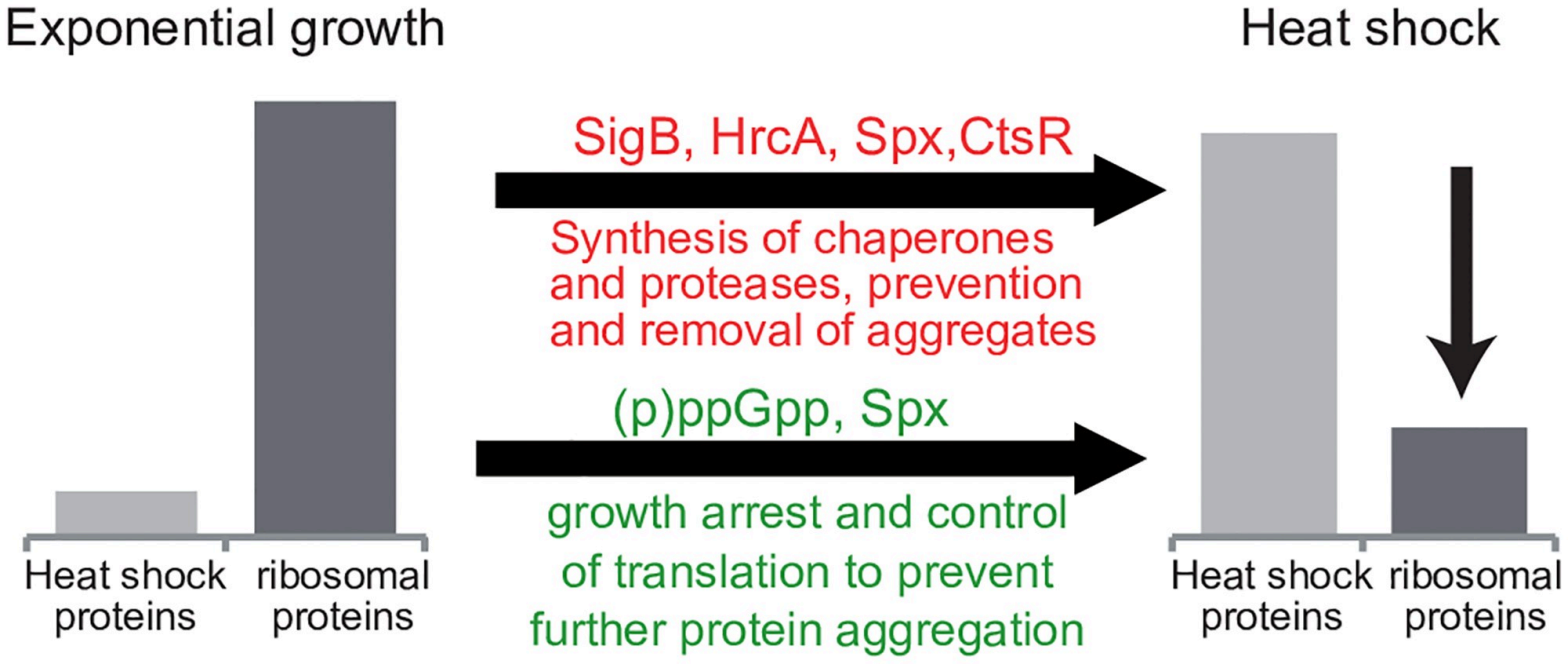
The role of the stringent response in the heat shock response. Model of the role of the stringent response in the regulatory network of the heat shock response.

Taken together, our data suggest a model in which cells respond to heat-mediated protein unfolding and aggregation, not only by raising the repair capacity, but also by decreasing translation to concurrently reduce the load on the cellular protein quality control systems (Fig 7).

Upon heat shock, Rel is activated and rapidly synthesizes alarmones. The second messenger (p)ppGpp can then directly control the activity of translation factors and may thereby mediate a fast and immediate response to modulate translation during stress. At the same time (p)ppGpp might play a significant role in maintaining and protecting active ribosomes, which might involve modulating translation already at the ribosome assembly stage. The readjustments of translation could then allow an efficient reallocation of cellular resources to the synthesis of stress response proteins and concurrently minimize the load on the protein quality control systems, thus contributing to protein homeostasis [3,82,83]. The unfolded protein response to misbalances in protein homeostasis in the endoplasmic reticulum of eukaryotic cells is a well-studied and analogous stress response mechanism where the up-regulation of chaperones is also coupled to the concurrent down-regulation of translation, albeit by different mechanisms [3,84].

It should be noted that lowering the cellular GTP level by treatment with decoyinine also resulted in a limited increase of thermoresistance in the absence of elevated alarmone levels. Similarly, it was reported that mutant strains of *Lactococcus lactis* with constitutively lowered GTP levels also exhibited increased stress tolerance [85]. These observations suggest, that a decrease in the cellular GTP concentration alone can reproduce many effects on the cellular physiology that can also be observed in the presence of (p)ppGpp. However, the heat stress resistance conferred by increased (p)ppGpp levels appeared to be stronger than observed upon decoyinine treatment, which could indicate that certain processes important for stress resistance are predominantly controlled by (p)ppGpp directly. Clearly, more work is required to identify and characterize the cellular targets of (p)ppGpp and to examine the differential roles of GTP and (p)ppGpp during thermotolerance development.

Interestingly, accumulation of (p)ppGpp upon heat or oxidative stress and its importance for stress resistance has also been reported in other *Firmicutes* and also *Proteobacteria* that differ widely in terms of (p)ppGpp signaling [49,74,77,86,87]. Accumulation of (p)ppGpp was shown to protect cells from salt or osmotic stress [85,88]. Conversely, the lack of (p)ppGpp is known to render cells sensitive to heat or oxidative stress [74,89,90], suggesting that activation of the SR, allowing the fast down-regulation of translation, is an important and conserved part of the response to environmental stress in bacteria. It is interesting to note that the SR was also implicated in *B*. *subtilis* competence development, facilitating a cellular state where cells cease to divide, and most transcription and translation is strongly down-regulated (also referred to as the K-state). In these cells, only competence proteins, together with DNA repair and recombination genes, are expressed, allowing the uptake and possible utilization of homologous DNA in this specific cellular state of a subpopulation of stationary phase cells [80]. Bacterial cells thus appear to utilize the (p)ppGpp second messengers, which can interfere directly with basic cellular processes such as translation, replication and growth, as an important part of different regulatory networks, facilitating and allowing the survival of bacterial cells in fast changing environments with limited nutrient availability and exposure to various stress conditions.

## Methods

### Construction of strains and plasmids

Strains, plasmids and primers are listed in S1 Table. PCR-amplification and molecular cloning using *E*. *coli* DH5α as host was carried out according to standard protocols [91]. Point mutations were introduced via overlap-extension PCR. To generate pBSII-spxDD-spec, a fragment carrying *spx*^*DD*^, *lacI* and the spectinomycin resistance cassette was amplified from pSN56 [15] with primers p289/p223 and ligated using *Spe*I/*Nsi*I sites into the pBSIIE backbone amplified with primers p203/p288. Integrative plasmids were linearized by digestion with *Sca*I or *Bsa*I prior to transformation. Point mutations in the *rel* gene were first cloned in the pMAD vector and then re-amplified for cloning into pDR111.

Transformation of *B*. *subtilis* strains, the generation of scarless mutations using the pMAD system and the introduction of *cxs-1/2* mutations in *rpoA* was carried out as described previously [92–94]. Mutants were selected on 100 μg ml^-1^ spectinomycin, 10 μg ml^-1^ kanamycin, 1 μg ml^-1^ erythromycin, 25 μg ml^-1^ lincomycin or 5 μg ml^-1^ chloramphenicol, respectively. To obtain the (p)ppGpp° strain (BHS214), markerless *relP*^E154V^ and *relQ*^E139V^ mutations were introduced into *B*. *subtilis 168* cells by successive transformation and recombination of plasmids pMAD-relP^E154V^ and pMAD-relQ^E139V^, yielding strain BHS204. Next, a PCR amplified fragment carrying *rel*::*erm* [27] and flanking homologous regions was transformed to generate BHS214. Since the (p)ppGpp° strain fails to develop natural competence, additional mutations were introduced in BHS204 and transformed with a PCR-amplified *rel*::*erm* fragment or BHS214 genomic DNA in a second step.

### Growth conditions

*B*. *subtilis* strains were grown in LB medium (5 g L^-1^ yeast extract, 10 g L^-1^ tryptone-peptone, 10 g L^-1^ NaCl) or minimal medium [95] supplemented with 0.5% casamino acids in water baths with 200 rpm orbital shaking at the desired temperatures. 1 mM IPTG or 0.4% xylose was supplemented if required.

### Survival and viability assays

The assays for thermotolerance development, survival and preparation of protein aggregate are described previously [9]. 1 mM IPTG was added to induce expression of recombinant proteins 30 min before the division of the culture. The influence of decoyinine on thermotolerance was tested in 1.5 mL tubes in a ThermoMixer (Eppendorf). Detection of aggregates by fluorescence microscopy was described previously in [48]. Spot colony formation assays were carried out as described previously and incubated at the indicated temperatures [17].

### Transcription analysis

Strains were grown in LB and treated as indicated. Samples of 15–25 mL were harvested by centrifugation for 3 min at 3,860 x g at 4 °C and frozen in liquid nitrogen. Isolation of total RNA, treatment with DNase I (NEB) and quality control by native agarose gel electrophoresis, methylene blue staining and northern blotting was described previously [17]. Northern blotting, hybridization with DIG-labeled RNA probes and detection was carried out as described previously [17]. Primers for the synthesis of probes are listed in S1 Table. Reverse transcription and qPCR were carried out as described previously [17]. The primers are listed in S1 Table. 23 S rRNA was used as a reference.

### RNA sequencing

Cells of BHS220, BHS319 and BHS368 were grown in 150 mL LB medium in 500 mL flasks in water baths at 37 °C and 200 rpm. In the mid-exponential phase (OD_600 nm_ ~ 0.4), the culture was divided and shifted to 48 °C or left at 37 °C. After 15 min, samples were withdrawn and both cultures were shifted to 53 °C for another 15 min and harvested. Cells from 25 mL medium were pelleted by centrifugation for 3 min at 3,860 x g and 4 °C and flash-frozen in liquid nitrogen. RNA was prepared the using phenol/trizol method as described in [96] and treated with TURBO DNase (Invitrogen). RNA quality was assessed on a Bioanalyzer 2100 System (Agilent).

rRNA depletion from total RNA using MICROBExpress (Ambion), treatment with tobacco acid pyrophosphatase (TAP) for +TAP libraries, library preparation, Illumina sequencing and quality control of the sequencing output was carried out as described previously [97]. Reads were mapped to the *Bacillus subtils* 168 genome with insertion of *rrnJp1-lacZ* in the *amyE* site (strain BHS220, *amyE*::*rrnJp1-lacZ cat*) using Bowtie2 (version 2.1.0) reads [98] with default parameters and filtered for uniquely mapped reads using SAMtools [99]. The DEseq2 package with default parameters was used for the detection of differentially expressed genes from raw count data of triplicate experiments [100]. Expression changes were considered significant if differentially regulated by at least 4-fold (*p*-value ≤ 0.05). The data have been deposited in NCBI’s Gene Expression Omnibus and are accessible through GEO Series accession number GSE125467 [101]. Transcription start sites were annotated from the comparison of rRNA-depleted, tobacco acid pyrophosphatase (TAP) treated libraries that allow adaptor-ligation to 5’ primary transcripts and libraries, where TAP treatment was omitted using the TSSpredator v1.06 software [102] in the “more sensitive” parameter preset and manually reviewed.

### *In vitro* transcription

*In vitro* transcription assays using purified *B*. *subtilis* RNA polymerase and Spx protein was carried out as described previously [17].

### Fluorescence microscopy

Strain BIH369 (*lacA*::*Pxyl-yocM-mCherry erm*) was grown in LB medium + 0.5% xylose. The culture was divided in the mid-exponential phase, supplemented with puromycin for 15 min and subjected to fluorescence microscopy in a Axio Imager.Z2 (Zeiss) microscope using the RFP filter set [17].

### SDS PAGE and western blotting

Strains were grown in LB medium and treated as indicated, harvested by centrifugation for 5 min at 3,860 x g at 4 °C, washed in TE buffer (10 mM TRIS-HCl, 1 mM EDTA, pH 8.0) and disrupted by sonication in TE supplemented with 1 mM PMSF. Equal amounts of protein were separated by SDS-PAGE and stained with coomassie or subjected to western blotting [103–105]. For signal detection, polyclonal α-Hpf antibody (1:5,000) [71] or monoclonal anti-puromycin antibody (1:10,000, Merck) and HRP-conjugated anti-mouse or anti-rabbit antibodies (1:10,000, Roth) were used in conjunction with the ECL-system as described previously [17]. Images were acquired using a ChemoStar Imaging System (Intas, Göttingen, Germany).

### Translation rate analysis

Puromycin becomes covalently incorporated into nascent peptide chains, which can be used as readout for the rate of translation [67,106,107]. We verified that low puromycin concentrations (1 μg mL^-1^) do not perturb growth or lead to the accumulation of misfolded proteins in cellular protein aggregates, which can be visualized as using the previously established YocM-mCherrry fusion protein (S10A–S10C Fig) [48,108]. Strains were grown in LB medium and treated as indicated. For *in vivo* labeling, 10 mL medium were separated, supplemented with 1 μg mL^-1^ puromycin (Roth) and incubated for 15 min at the same conditions. Then, samples were supplemented with 25 μg mL^-1^ chloramphenicol, harvested by centrifugation for 5 min at 3,860 x g at 4 °C, washed in TE buffer (10 mM TRIS-HCl, 1 mM EDTA, pH 8.0) and disrupted by sonication in TE supplemented with 1 mM PMSF. Equal amounts of protein were directly spotted on nitrocellulose membranes (5 μg) or subjected to SDS-PAGE and western blotting [91]. Puromycin-signals were detected using monoclonal anti-puromycin antibody (1:10,000, Merck), HRP-conjugated anti-mouse antibody (1:10,000, Roth) and the ECL-system in a ChemoStar imaging system (Intas, Göttingen, Germany). Signals were analyzed using Fiji distribution of ImageJ [109].

### Sucrose density gradient centrifugation analysis

Early exponential phase cultures of *B*. *subtilis* strains grown in LB medium were treated with heat shock at 48 °C or 48 °C/53 °C for 15 min each. Samples of 50 mL were supplemented with 50 μg mL^-1^ chloramphenicol to stall translation and harvested by centrifugation at 4,000 x g for 10 min at 4 °C. Cells were resuspended in 25 mM HEPES-KOH, pH 7.5, 150 mM KOAc, 25 mM Mg(OAc)_2_, 1 mM dithiothreitol (DTT), n-Decyl−β−D-thiomaltopyranoside (DTM), 5% (w/v) sucrose) and lysed by sonication. The lysate was cleared by centrifugation at 16,000 x g for 15 min at 4 °C. 10 OD_260_ units were loaded on a 10 mL 5–45% (w/v) sucrose gradient prepared in the same buffer, run in a SW-40 Ti rotor (Beckman Coulter) at 57,471 x g for 16.5 h and analyzed using a Gradient Station (Biocomp) with an Econo UV Monitor (Bio-Rad).

### Quantification of nucleotides

Cells were grown in minimal medium supplemented with 0.5% casamino acids to support the growth of (p)ppGpp deficient strains [60] and treated as indicated. Samples of 2 mL were removed, supplemented with 75 μL 100% formic acid and incubated on ice for 30 min. Extraction of nucleotides was carried out as described in [110] and detected by HPLC-ESI-MS/MS on a QTRAP 5500 instrument. Analytes were separated on a Hypercarb column (30 x 4.6 mm, 5 μm particle size) in a linear gradient of solvent A (10 mM ammonium acetate pH 10) and solvent B (acetonitrile) at a flow rate of 0.6 mL/min from 96% A + 4% B (0 min) to 40% A + 60% B (8 min) into the ESI ion source at 4.5 kV in positive ion mode. Tenofovir was used as internal standard. pGpp and pppGpp standards were synthesized *in vitro* from ATP and GTP or GMP as described previously [111]. ppGpp was purchased from Trilink Biotechnologies.

### Identification and quantification of proteins by mass spectrometry

Strains were grown in LB medium at 37 °C and 200 rpm to the mid-exponential phase (OD_600nm_ 0.4) and transferred to a 50 °C water bath. Samples were taken at before, 5 min and 15 min after the temperature shift and washed three times in 50 mM HEPES pH 8, 150 mM NaCl. Three biological replicates were analyzed. All samples were subjected to SP3 sample preparation [112]. Briefly, to each sample 4x lysis buffer was added (4% SDS, 40 mM TCEP, 160 mM chloroacetamide, 200 mM HEPES pH 8) and proteins were denatured, reduced and alkylated during incubation at 95 °C for 5 minutes. 0.8 μL Benzonase (NEB) was added and samples were incubated at 37 °C for 30 minutes. Ten μg of a 1:1 mixture of hydrophilic and hydrophobic carboxyl-coated paramagnetic beads (SeraMag, #24152105050250 and #44152105050250, GE Healtcare) were added for each μg of protein. Protein binding was induced by addition of acetonitrile to a final concentration of 50% (v/v). Samples were incubated for 10 minutes at room temperature. The tubes were placed on a magnetic rack and beads were allowed to settle for three minutes. The supernatant was discarded and beads were rinsed three times with 200 μL of 80% ethanol without removing the tubes from the rack. Beads were resuspended in digestion buffer containing 50 mM triethylammonium bicarbonate and both Trypsin (Serva) and Lys-C (Wako) in a 1:50 enzyme to protein ratio. Protein digestion was carried out for 14 hours at 37°C in a PCR cycler. Afterwards the supernatant was recovered and 1 μL was used to perform peptide quantification using a quantitative colorimetric peptide assay (Pierce, #23275) following the manufacturer’s instructions.

TMT 11plex (Pierce, #A37725) was used for peptide multiplexing and quantification. Briefly, equal amounts of peptides were dried down in a vacuum concentrator and resuspended in 50 mM HEPES pH 8.5. Additionally, 10% from each sample was pooled to create a common sample as internal standard. TMT reagents were allowed to equilibrate to room temperature for 30 minutes and were dissolved in anhydrous acetonitrile to a final concentration of 59 mM. To each sample TMT was added to a final concentration of 11.8 mM and tubes were incubated at 25°C for 60 minutes with mixing at 500 rpm on a ThermoMixer. Labeling was quenched by addition of hydroxylamine to a final concentration of 0.4%. Samples were mixed, desalted using solid phase extraction (Seppak 1cc/50mg, Waters), dried down in a vacuum concentrator and resuspended in 20 μL 2% acetonitrile. Basic reversed phase fractionation was performed on a quaternary Agilent 1290 Infinity II UPLC system equipped with a Kinetex Evo-C18 column (150 x 2.1 mm, 2.6μm, 100 Å, Phenomenex) that was operated at 40 °C. Solvent A consisted of water, solvent B consisted of 100% acetonitrile, and solvent C consisted of 25 mM ammonium bicarbonate. Fractionation was carried out at a constant flow rate of 100 μl/min using a linear gradient from 2–25% acetonitrile within 50 minutes, followed by column washing and equilibration. Over the whole gradient solvent C was kept constant at 10%. In total 32 fractions were collected in conical 96well plates. The organic solvent was removed in a vacuum concentrator for one hour and fractions were combined into 8 final samples. Peptides were acidified with formic acid, desalted using OASIS HLB 96well cartridges (Waters, #186001828BA), dried down and resuspended in 2% acetonitrile, 0.1% trifluoroacetic acid (TFA) prior MS analysis.

All samples were analyzed on a Q-Exactive HF (Thermo Scientific) that was coupled to a 3000 RSLC nano UPLC (Thermo Scientific). Samples were loaded on a pepmap trap cartridge (300 μm i.d. x 5 mm, C18, Thermo) with 2% acetonitrile, 0.1% TFA at a flow rate of 20 μL/min. Peptides were separated over a 50 cm analytical column (Picofrit, 360 μm O.D., 75 μm I.D., 10 μm tip opening, non-coated, New Objective) that was packed in-house with Poroshell 120 EC-C18, 2.7 μm (Agilent). Solvent A consists of 0.1% formic acid in water. Elution was carried out at a constant flow rate of 250 nL/min using a 180 minute method: 8–33% solvent B (0.1% formic acid in 80% acetonitrile) within 120 minutes, 33–48% solvent B within 25 minutes, 48–98% buffer B within 1 minute, followed by column washing and equilibration. Data acquisition on the Q-Exactive HF was carried out using a data-dependent method in positive ion mode. MS survey scans were acquired from 375–1500 m/z in profile mode at a resolution of 120,000. AGC target was set to 3e6 charges at a maximum injection time of 60 ms. The ten most abundant peptides were isolated within a 0.7 m/z window offset by +0.1 m/z and subjected to HCD fragmentation at a normalized collision energy of 32%. The MS2 AGC target was set to 2e5 charges, allowing a maximum injection time of 78 ms. Product ions were detected in the Orbitrap at a resolution of 45,000. Precursors were dynamically excluded for 30 s.

Raw files were processed with Proteome Discoverer 2.3 (Thermo Scientific). Briefly, peak lists were extracted from raw files and searched using SEQUEST HT against a Uniprot bacillus subtilis database (version 190614, taxonomy ID 224308) and a database containing sequences of common contaminants (derived from Maxquant v.1.6.0.1). Trypsin/p was set as enzyme specificity, allowing a maximum of two missed cleavages. The minimum peptide length was set to 7 amino acids. Carbamidomethylation on cysteine was set as fixed modification. Protein N-terminal acetylation, oxidation of methionine, and TMT on lysines and peptide n-termini were allowed as variable modifications. Mass tolerances for MS1 and MS2 were set to 10 ppm and 0.02 Da, respectively. Peptide-spectrum-matches (PSMs) were filtered to a 1% FDR level using Percolator employing a target/decoy approach. Only rank 1 peptides were allowed. TMT reporter ion intensities were quantified within 20 ppm windows and quan value correction was used to correct for reagent isotope impurities. Only unique peptides were used for protein quantification. PSM with a co-isolation value of >50% were rejected. Further data processing was carried out in R and Perseus (v. 1.6.2.3). Only proteins identified with at least two peptides were included in the analysis. All contaminant proteins and proteins that have not been quantified in all 18 samples were filtered out. A three step normalization procedure was applied. First, the sum of the reporter ion intensities for each TMT channel was normalized to the average grand total to correct for mixing errors. Next, the common internal standard in each TMT 11plex set was used for internal reference scaling [113] in order to correct for batch effects. Afterwards the data was normalized applying trimmed mean of M values (TMM) using the edgeR package. Statistical analysis was performed using two-sample t-tests and multiple sample ANOVA tests. Resulting p-values were corrected for multiple testing using a permutation-based FDR approach or by the method of Benjamini-Hochberg. The mass spectrometry proteomics data have been deposited to the ProteomeXchange Consortium via the PRIDE [114] partner repository with the dataset identifier PXD015416.

### Gene set enrichment analysis

A gene set enrichment analysis (GSEA) of the significantly regulated genes or proteins was carried out on the Category (SW1 to SW4) and regulon datasets provided by SubtiWiki (http://subtiwiki.uni-goettingen.de/v3/category/) [115]. The GNU R software v. 3.5.1 [116] and the clusterProfiler library v. 3.10.1 [117] was used. P values were adjusted according to the Benjamini-Hochberg (BH) method and P_adjust_ ≤ 0.05 was set as significance threshold.

## Supporting information

S1 Fig Alarmone and GTP levels during stress and starvation. (A) Means and SEM of GTP after the application of different stress conditions. Sample sizes and treatments are the same as in Fig 1A and 1B. NV: DL-norvaline, SHX: serine hydroxamate. Asterisks (*) indicate significance (*p*_*adj*._ ≤ 0.05) of combined pGpp, ppGpp and pppGpp levels according to the Kruskal-Wallis and Dunn-Bonferroni test. (B) Levels of GTP during thermotolerance development. Wild type cells were grown at 37 °C and shifted to 48 °C for 15 min (pre-shock), then to 53 °C or directly to 53 °C. Samples were taken at 2, 5 and 15 min. Means and SEM of four independent experiments are shown. All changes are not significant (p ≤ 0.05) according to the Kruskal-Wallis test. (C) Means and SEM of GTP levels in wild type cells or strains with mutations in (p)ppGpp synthetases (*relP/Q*^-^: BHS204, *rel*^E324V^ (inactive synthetase): BHS709; (p)ppGpp°: BHS214) treated with heat stress (2 min 50 °C) or left untreated at 37 °C. Sample sizes are the same as in Fig 1E. Asterisks indicate significant changes (*p* ≤ 0.05) according to Welch’s *t*-test. (D) Relative changes in the transcription of *hpf* during heat shock in the same strains (15 min 50 °C). Means and SEM of three independent experiments are shown. Asterisks (*) indicate significant changes (*p*_*adj*._ ≤ 0.05) according to the Kruskal-Wallis and Dunn-Bonferroni test. (E) The influence of chloramphenicol on GTP levels during stress. Sample sizes and treatments are the same as in Fig 1F. Asterisks indicate significant changes (*p* ≤ 0.05) according to Welch’s *t*-test.
(TIFF)

S2 Fig Phenotype of single deletions of (p)ppGpp synthetase genes. **(A)** Cellular GTP levels in wild type or Δ*rel*: (BHS126) strains. **(B)** Growth of strains with mutations or deletions in (p)ppGpp metabolizing enzymes in rich LB medium. Δ*rel*: BHS126, (p)ppGpp°: BHS214. **(C/D)** Survival of wild type (black lines) and mutant strains (Δ*relQ*: BHS127 or Δ*relP*: BHS128) red lines at 53 °C with (48/53 °C) or without (37/53 °C) pre-shock. Means and SEM of at least three independent experiments are shown. Open symbols: no pre-shock, closed symbols: 15 min pre-shock at 48 °C. **(E)** Growth of wild type, (p)ppGpp° cells (BHS214) or strains with deletions in *relQ* (BHS127) or *relP* (BHS128) on agar plates at 37 °C, during heat stress (55 °C) or oxidative stress (0.2 mM diamide).
(TIFF)

S3 Fig Thermotolerance and survival of strains expressing *rel* variants *in trans*. **(A)** Levels of alarmones in strains expressing hyperactive or inactive variants of *B*.*s*. *rel* (E324V: inactive synthetase, E77A/D78A: inactive hydrolase) or *E*.*c*. *relA*. Cells were treated with 1 mM IPTG for 15 min. Asterisks indicate significant changes (*p* ≤ 0.05) of combined alarmone levels according to Welch’s *t*-test. **(B-E)** Survival of wild type (black lines) and mutant strains (red lines) at 53 °C without pre-shock (37/53 °C; open symbols) or with pre-shock (15 min 48 °C/53 °C; closed symbols). Means and SEM of at least three independent experiments are shown. Strains were supplemented with 1 mM IPTG 15 min prior to 48 °C temperature shift. **(B)** Expression of a truncated, hyperactive *E*. *coli relA* variant (designated *relA*_*hyper*_). **(C)** Expression of *B*.*s*. *rel* with inactive hydrolase domain (E77A D78A) in the (p)ppGpp° strain. **(D)** Expression of a truncated, inactive *E*. *coli relA* variant (*relA*_*inactive*_). (E) Expression of *B*.*s*. *rel* with inactive synthetase domain (E324V) in the (p)ppGpp° strain.
(TIFF)

S4 Fig Thermotolerance and survival of strains expressing treated with decoyinine. Thermotolerance development and survival of wild type cells treated with decoyinine (red lines) or left untreated (black lines).
Means and SEM of at least three independent experiments are shown. Strains were supplemented with 50, 250, 400 or 1000 μg ml^-1^ decoyinine 15 min before heat treatment. Open symbols: no pre-shock, closed symbols: 15 min pre-shock at 48 °C. n.d.: not determined, no cfu could be detected from 100 μl cell culture.
(TIFF)

S5 Fig Transcriptional changes mediated by changed (p)ppGpp levels or heat stress. **(A)** Comparison of the relative transcription changes of selected genes in, Δrel and (p)ppGpp0 strains during exponential growth at 37 °C as determined by RNA-seq or RT-qPCR from in-dependent experiments. Means and SEM of three replicates are shown. **(B)** Heatmap showing the expression changes of selected transcripts in wild type, (p)ppGpp° or Δ*rel* strains. Values represent normalized log_2_ scaled read counts centered on the mean expression level of each transcript. **(C/D)** The distributions of all up- and down-regulated genes in wild type cells (BHS220) heat shocked at 48 °C or 53 °C versus unstressed cells are shown. Bar tracks indicate the distribution of the respective functional groups.
(TIFF)

S6 Fig Up- or down-regulation of regulons or gene categories. Points in the scatterplot represent log2-transformed up- or down-regulation of individual genes of the respective regulons relative to wild type cells at 37 °C. Blue/gray color indicates transcriptional changes above/below the significance threshold (see Materials and Methods). Horizontal bars represent the median expression changes of the whole gene set.
(TIFF)

S7 Fig (p)ppGpp mediated transcriptional changes during heat stress. **(A)** Relative changes in the transcription of selected genes known to controlled by the stringent response during heat shock in wild type and (p)ppGpp° strains determined by RT-qPCR. Means and SEM of three replicates are shown. Asterisks indicate significance (p ≤ 0.05) according to Welch’s t-test. **(B/C)** Heatmap showing expression changes of selected transcripts during mild heat stress in wild type, (p)ppGpp° or Δ*rel* cells. Values represent log_2_ fold changes of transcript levels relative to wild type cells at 37 °C. **(D)** Relative changes in the transcription of selected stress response genes during heat shock in wild type and (p)ppGpp° strains determined by RT-qPCR. Means and SEM of three replicates are shown. Asterisks indicate significance (p ≤ 0.05) according to Welch’s t-test.
(TIFF)

S8 Fig Phenotypes of (p)ppGpp°, Δ*spx* and (p)ppGpp° Δ*spx* strains. **(A)** RT-qPCR experiment showing the relative transcription of *rplC* and *rplO* in wild type (BHS220), Δ*spx* (BHS222), (p)ppGpp° (BHS319) or (p)ppGpp° Δ*spx* (BHS766) cells treated with or heat stress at 50 °C for 15 min. Means and SEM of three replicates are shown. Asterisks indicate significant changes (*p* ≤ 0.05) of transcript levels according to Welch’s *t*-test. **(B)** Growth of wild type, Δ*spx*, (p)ppGpp° or (p)ppGpp° Δ*spx* cells in LB medium at 37 °C (left) as well as growth of wild type, (p)ppGpp°, *cxs*-1, *cxs*-2, (p)ppGpp° cx*s*-1 or (p)ppGpp° cx*s*-2 cells in LB medium at 50 °C (right). **(C**) The fraction of aggregated proteins (left) or soluble proteins (right) in wild type, Δ*spx* (BHS014), (p)ppGpp° (BHS214) or (p)ppGpp° Δ*spx* (BHS766) cells treated with or heat stress at 50 °C for 15 min.
(TIF)

S9 Fig (p)ppGpp and Spx act independently. **(A)** Northern and western blot of wild type, Δ*spx* (BHS014) or (p)ppGpp° (BHS214) strains treated with or without DL-norvaline. Cells were grown in minimal medium supplemented with 0.5% casamino acids to OD_600_ 0.4. The medium was removed by centrifugation and the cells were resuspended in fresh medium with casamino acids (—) or 0.5 mg/ml DL-norvaline (+) and grown for 30 min. **(B)** Relative transcription of *rrnJp1-lacZ* with or without expression of *spx*^*DD*^ with 1 mM IPTG for 30 min in the wild type or (p)ppGpp° background as determined by RT-qPCR. Means and SEM of three replicates are shown. Asterisks indicate significant changes (*p* ≤ 0.05) of transcript levels according to Welch’s *t*-test. **(C)** *In vitro* transcription experiments with selected promoters in the presence or absence of Spx or ppGpp under reducing (+ DTT) or oxidizing (- DTT) conditions. Means and SEM of three replicates and a representative autoradiogram are shown.
(TIFF)

S10 Fig Puromyin labels nascent proteins and does not disturb protein homeostasis at low concentration. **(A)** Accumulation of subcellular protein aggregates (fluorescent spots) after the addition of puromycin visualized by YocM-mCherry. BIH369 cells were grown in LB + 0.5% xylose and treated with 1, 10 or 25 μg ml^-1^ puromycin or left untreated for 15 min. Phase contrast images (P.C.) and fluorescence images with RFP-filters (YocM-mCherry) are shown. **(B)** The effect of puromycin on growth. Wild type cells were grown in LB to the mid-exponential phase (OD_600_ 0.4) and supplemented with puromycin at the indicated concentrations. **(C)** Dot blot or western blot of puromycin-labeled proteins. Exponentially growing cells grown in LB were treated with the indicated concentrations of puromycin for 15 min. **(D)** Outline of the genotypes of the RIK1066 strain, carrying an inducible copy of *relP* in the (p)ppGpp° background. **(E)** Relative puromycin incorporation in RIK1066 cells treated with or without 1 mM IPTG. Cells were incubated with 1 mg ml^-1^ puromycin for 15 min added directly to the medium after the addition of IPTG (0–15 min) or after 15 min (15–30 min), then harvested. One representative experiment and means and SEM from the quantification of three independent experiments are shown. Asterisks indicate significance (*p* ≤ 0.05) according to Welch’s *t*-test.
(TIFF)

S11 Fig Growth and thermotolerance development of Δ*rplK* cells. **(A)** Growth of wild type and Δ*rplK* (BHS859) cells. **(B)** Relative translation rates of wild type and Δ*rplK* (BHS859) cells at 37 °C. **(C)** Thermotolerance development and thermoresistance of Δ*rplK* (BHS859) cells. Means and standard error of three biological replicates are shown.
(TIFF)

S12 Fig The influence of (p)ppGpp on individual protein levels upon heat treatment. Levels of individual proteins in wild type and mutant strains with or without heat treatment (50 °C for 5 min or 15 min at 50 °C) relative to unstressed wild type cells. Means and standard error of three biological replicates are shown.
(TIFF)

S13 Fig Global changes in the proteome mediated by heat shock or (p)ppGpp. The distributions of all up- and down-regulated in wild type or mutant cells with or without heat treatment (15 min 50 °C). Bar tracks indicate the distribution of the respective functional groups.
(TIFF)

S14 Fig The role of (p)ppGpp and Hpf on ribosome integrity and 100S formation. **(A)** Relative translation (puromycin incorporation) of wild type, (p)ppGpp° (BHS214) and Δ*rel* (BHS126) strains during heat stress at 53 °C. 1 μg ml^-1^ puromycin was added for 15 min to the medium directly after (sample “0–15 min”) or 15 min (sample “15–30 min”) after the temperature upshift. Means and SEM of three independent experiments are shown. Asterisks indicate significance (*p* ≤ 0.05) according to Welch’s *t*-test. **(B)** Western blot showing Hpf levels during thermotolerance development in wild type, (p)ppGpp° (BHS214) or Δ*rel* (BHS126) strains. Cells were heat shocked for 15 min each at the indicated temperature(s). **(C)** Sucrose gradient profiles of extracts from untreated (37 °C) or thermotolerant (48/53 °C for 15 min each) Δ*hpf* (BHS008) or Δ*rel* (BHS126) cells. The dashed blue line of untreated wild type cells is shown for reference. **(D)** Methylene blue stained membranes showing the integrity or degradation of rRNA. Wild type, (p)ppGpp° (BHS214) Δ*hpf* (BHS008) or (p)ppGpp° P_spac_-*hpf* (BHS626) cells were treated with or without heat shock at 53 °C for 15 min. 1 mM IPTG was added to the strains to induce the expression of *hpf* 15 min prior to heat shock. 2 μg total RNA was separated on denaturing agarose gels and blotted on nylon membranes. **(E)** Wild type, (p)ppGpp° (BHS214) (p)ppGpp° P_spac_-*rel* (BHS622) or (p)ppGpp° P_spac_-*hpf* (BHS626) were spotted on agar plates supplemented with 1 mM IPTG and incubated over night at 37 °C or 55 °C. **(F)** rRNA degradation after severe heat stress (53 °C) in wild type or (p)ppGpp° (BHS214) cells left untreated or treated with 5 μg ml^-1^ chloramphenicol or 100 μg ml^-1^ spectinomycin 15 min prior to the application of stress. 2 μg total RNA was separated on denaturing agarose gels and blotted on nylon membranes.
(TIFF)

S1 Table List of strains, plasmids and oligonucleotides. This table lists all *B*. *subtilis* strains, plasmids and oligonucleotides used in this study.
(DOCX)

S1 Dataset List of identified transcription start sites. In this dataset, all identified transcriptional start sites and their classification is shown.
(XLSX)

S2 Dataset Results of the gene set enrichment analysis. This dataset lists all enriched functional categories and regulons for each condition in separate sheets.
(XLSX)

S3 Dataset List of differentially expressed genes. Global gene expression changes for all conditions are listed in separate sheets.
(XLSX)

S4 Dataset List of differentially regulated proteins. Changes in the cellular levels of individual proteins in the examined strains and heat shock conditions.
(XLSX)

S5 Dataset Enrichment analysis for differentially expressed proteins. This dataset lists all enriched functional categories and regulons for all differentially expressed proteins of S4 Dataset.
(XLSX)
