# Supplementary figures and images for: The alarmones (p)ppGpp are part of the heat shock response of Bacillus subtilis
Source: PLoS Genet. 2020 Mar 16;16(3):e1008275. doi: 10.1371/journal.pgen.1008275 (PMC7098656; doi:10.1371/journal.pgen.1008275)

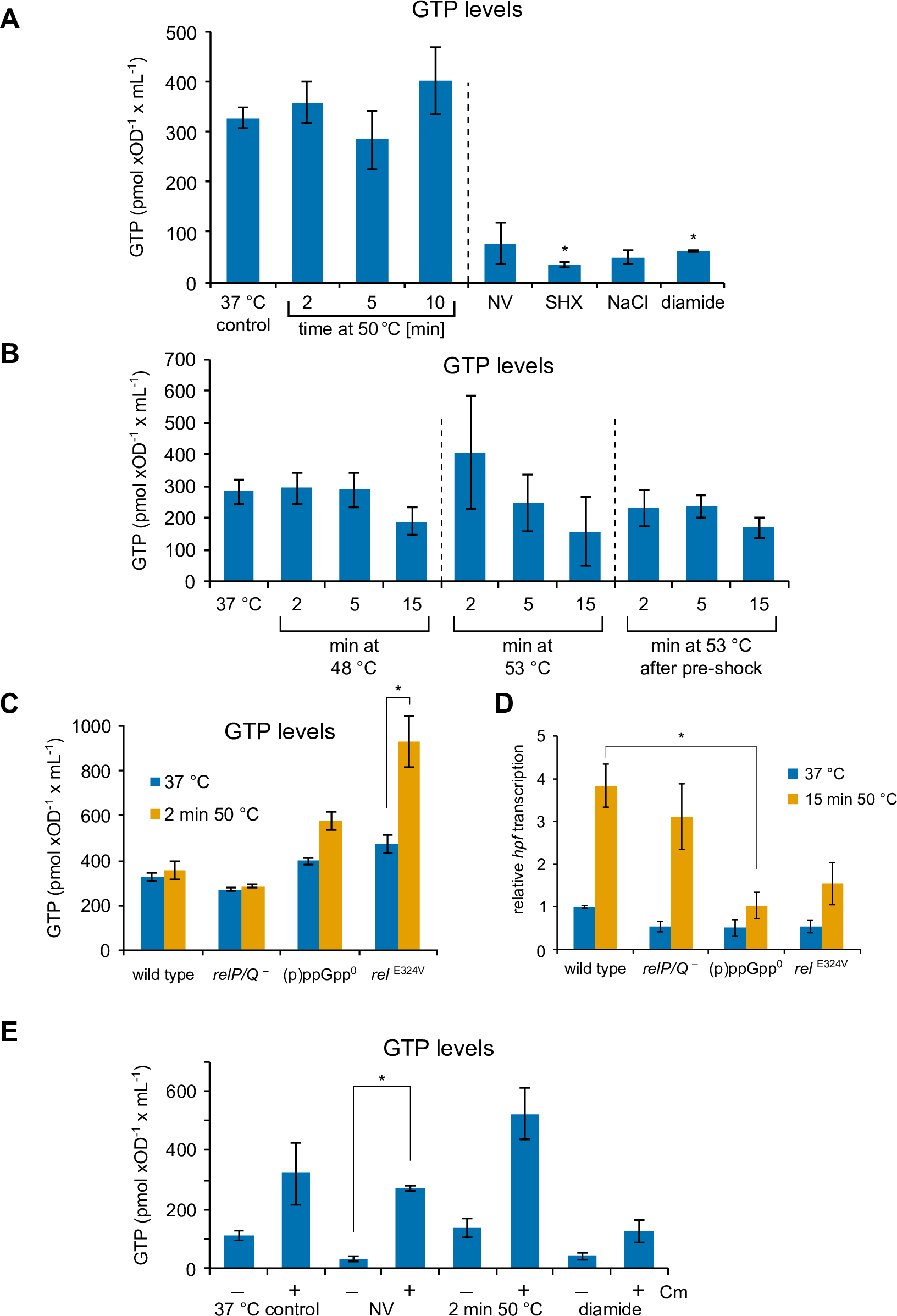

Supplement: S1 Fig — (A) Means and SEM of GTP after the application of different stress conditions. Sample sizes and treatments are the same as in Fig 1A and 1B. NV: DL-norvaline, SHX: serine hydroxamate. Asterisks (*) indicate significance (padj. ≤ 0.05) of combined pGpp, ppGpp and pppGpp levels according to the Kruskal-Wallis and Dunn-Bonferroni test. (B) Levels of GTP during thermotolerance development. Wild type cells were grown at 37 °C and shifted to 48 °C for 15 min (pre-shock), then to 53 °C or directly to 53 °C. Samples were taken at 2, 5 and 15 min. Means and SEM of four independent experiments are shown. All changes are not significant (p ≤ 0.05) according to the Kruskal-Wallis test. (C) Means and SEM of GTP levels in wild type cells or strains with mutations in (p)ppGpp synthetases (relP/Q-: BHS204, relE324V (inactive synthetase): BHS709; (p)ppGpp°: BHS214) treated with heat stress (2 min 50 °C) or left untreated at 37 °C. Sample sizes are the same as in Fig 1E. Asterisks indicate significant changes (p ≤ 0.05) according to Welch’s t-test. (D) Relative changes in the transcription of hpf during heat shock in the same strains (15 min 50 °C). Means and SEM of three independent experiments are shown. Asterisks (*) indicate significant changes (padj. ≤ 0.05) according to the Kruskal-Wallis and Dunn-Bonferroni test. (E) The influence of chloramphenicol on GTP levels during stress. Sample sizes and treatments are the same as in Fig 1F. Asterisks indicate significant changes (p ≤ 0.05) according to Welch’s t-test. (TIFF) [file pgen.1008275.s001.tiff]

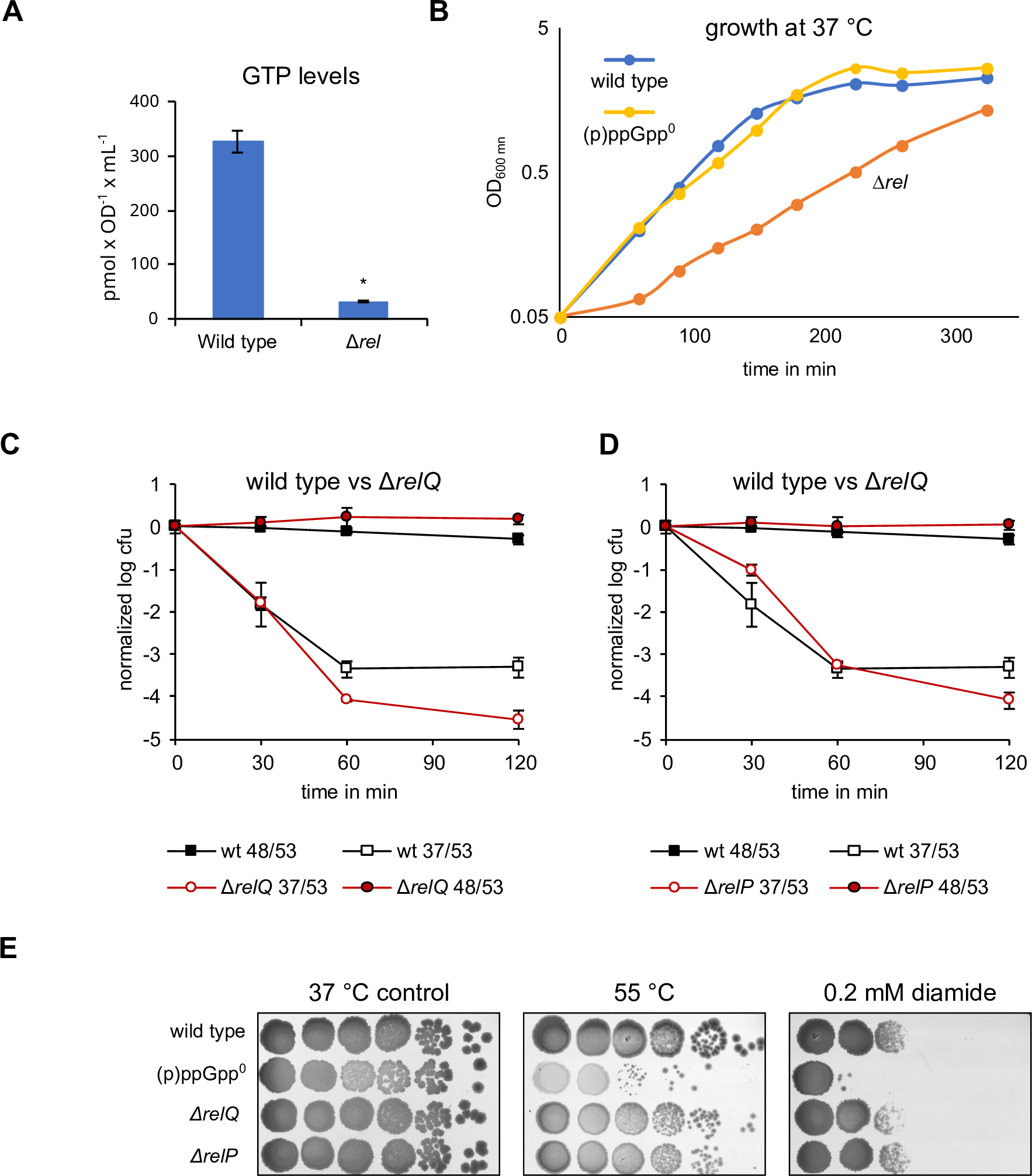

Supplement: S2 Fig — (A) Cellular GTP levels in wild type or Δrel: (BHS126) strains. (B) Growth of strains with mutations or deletions in (p)ppGpp metabolizing enzymes in rich LB medium. Δrel: BHS126, (p)ppGpp°: BHS214. (C/D) Survival of wild type (black lines) and mutant strains (ΔrelQ: BHS127 or ΔrelP: BHS128) red lines at 53 °C with (48/53 °C) or without (37/53 °C) pre-shock. Means and SEM of at least three independent experiments are shown. Open symbols: no pre-shock, closed symbols: 15 min pre-shock at 48 °C. (E) Growth of wild type, (p)ppGpp° cells (BHS214) or strains with deletions in relQ (BHS127) or relP (BHS128) on agar plates at 37 °C, during heat stress (55 °C) or oxidative stress (0.2 mM diamide). (TIFF) [file pgen.1008275.s002.tiff]

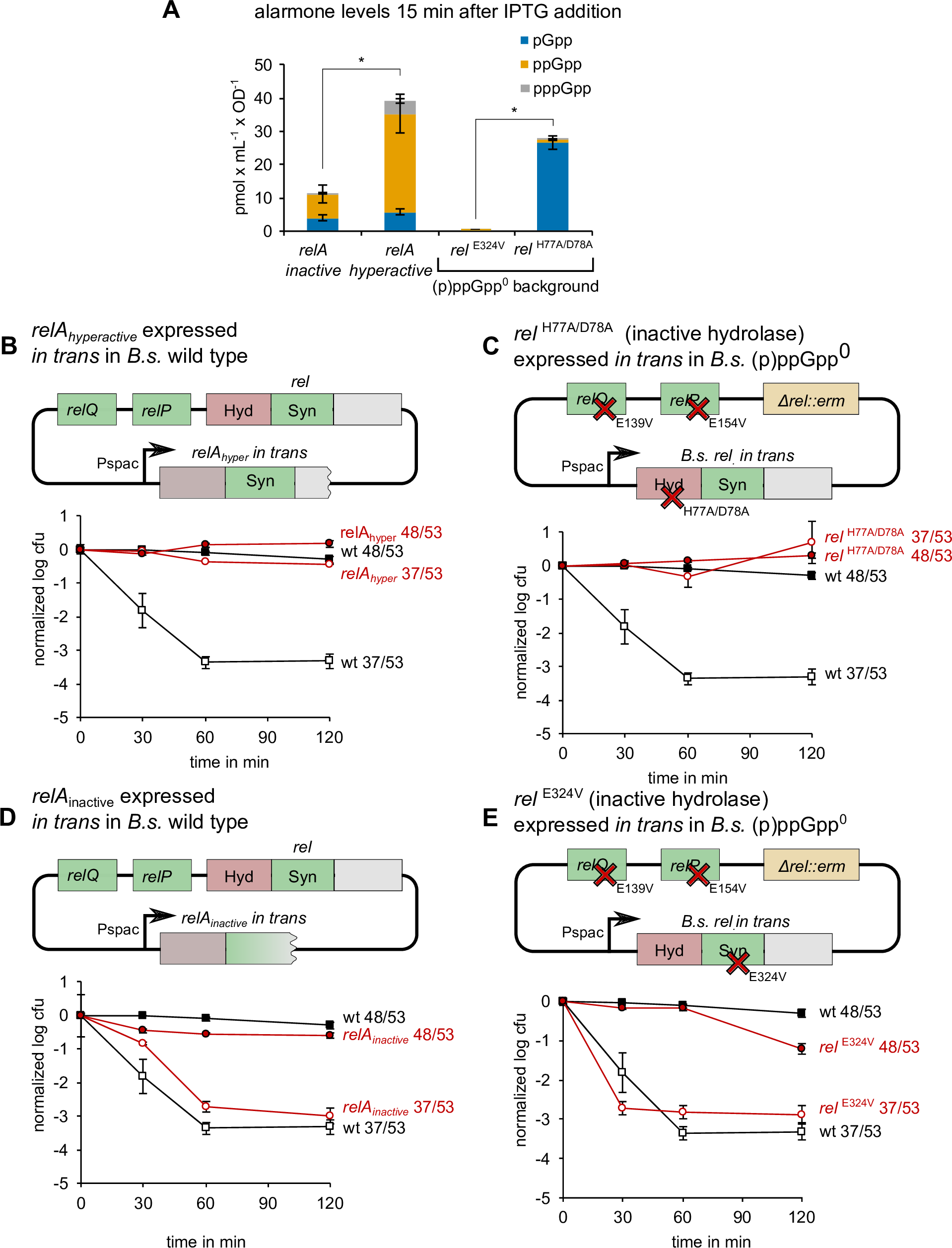

Supplement: S3 Fig — (A) Levels of alarmones in strains expressing hyperactive or inactive variants of B.s. rel (E324V: inactive synthetase, E77A/D78A: inactive hydrolase) or E.c. relA. Cells were treated with 1 mM IPTG for 15 min. Asterisks indicate significant changes (p ≤ 0.05) of combined alarmone levels according to Welch’s t-test. (B-E) Survival of wild type (black lines) and mutant strains (red lines) at 53 °C without pre-shock (37/53 °C; open symbols) or with pre-shock (15 min 48 °C/53 °C; closed symbols). Means and SEM of at least three independent experiments are shown. Strains were supplemented with 1 mM IPTG 15 min prior to 48 °C temperature shift. (B) Expression of a truncated, hyperactive E. coli relA variant (designated relAhyper). (C) Expression of B.s. rel with inactive hydrolase domain (E77A D78A) in the (p)ppGpp° strain. (D) Expression of a truncated, inactive E. coli relA variant (relAinactive). (E) Expression of B.s. rel with inactive synthetase domain (E324V) in the (p)ppGpp° strain. (TIFF) [file pgen.1008275.s003.tiff]

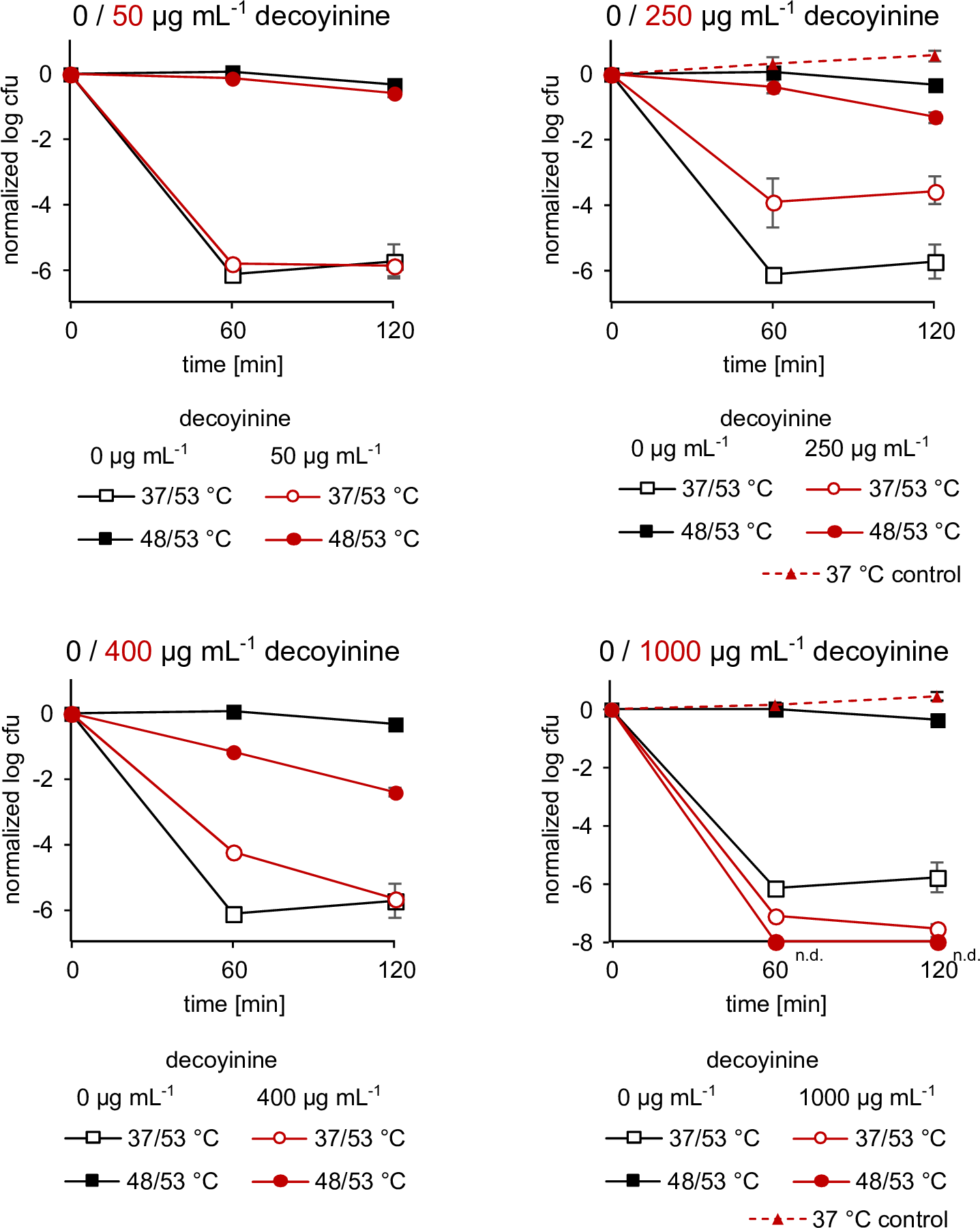

Supplement: S4 Fig — Thermotolerance development and survival of wild type cells treated with decoyinine (red lines) or left untreated (black lines). Means and SEM of at least three independent experiments are shown. Strains were supplemented with 50, 250, 400 or 1000 μg ml-1 decoyinine 15 min before heat treatment. Open symbols: no pre-shock, closed symbols: 15 min pre-shock at 48 °C. n.d.: not determined, no cfu could be detected from 100 μl cell culture. (TIFF) [file pgen.1008275.s004.tiff]

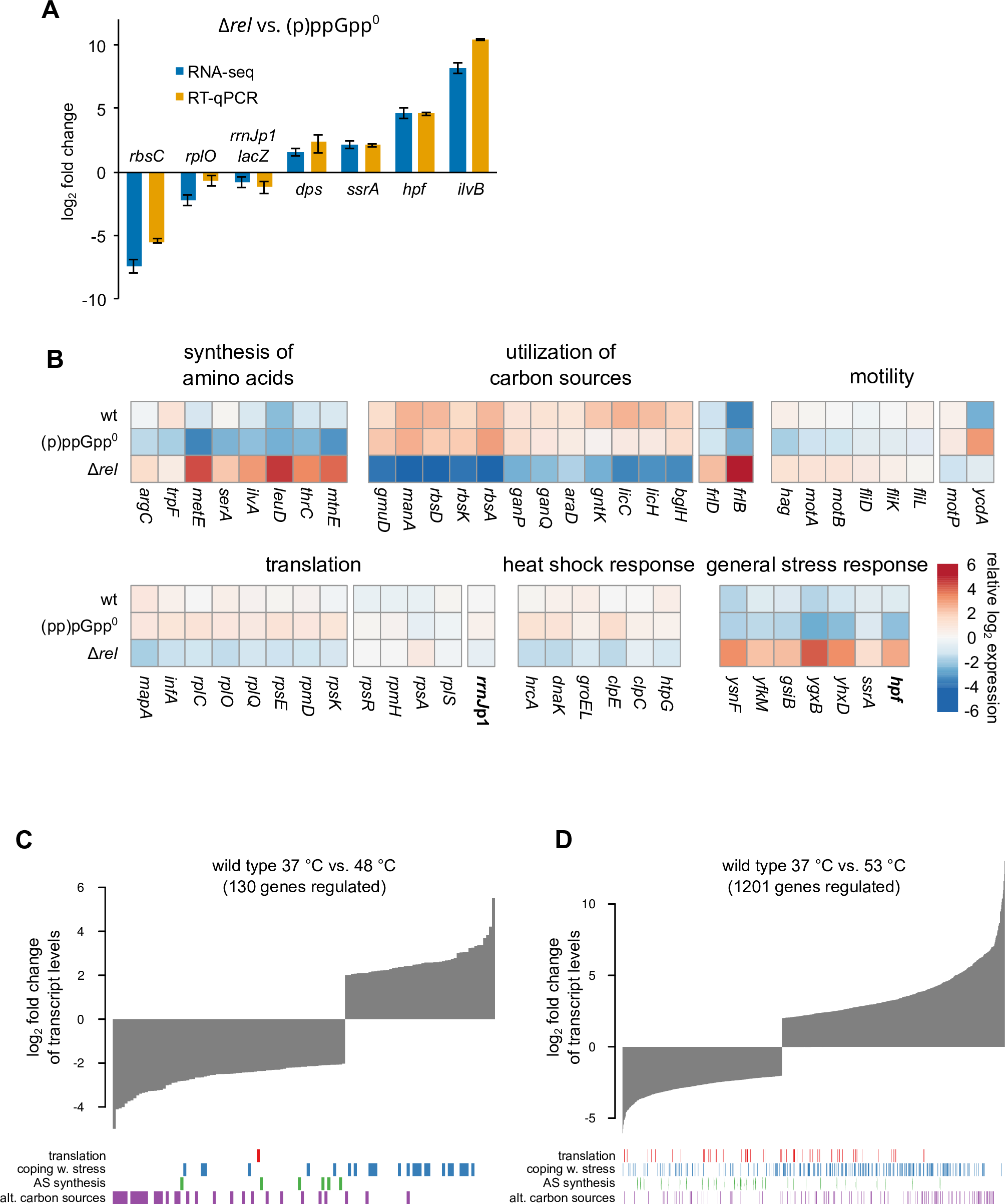

Supplement: S5 Fig — (A) Comparison of the relative transcription changes of selected genes in, Δrel and (p)ppGpp0 strains during exponential growth at 37 °C as determined by RNA-seq or RT-qPCR from in-dependent experiments. Means and SEM of three replicates are shown. (B) Heatmap showing the expression changes of selected transcripts in wild type, (p)ppGpp° or Δrel strains. Values represent normalized log2 scaled read counts centered on the mean expression level of each transcript. (C/D) The distributions of all up- and down-regulated genes in wild type cells (BHS220) heat shocked at 48 °C or 53 °C versus unstressed cells are shown. Bar tracks indicate the distribution of the respective functional groups. (TIFF) [file pgen.1008275.s005.tiff]

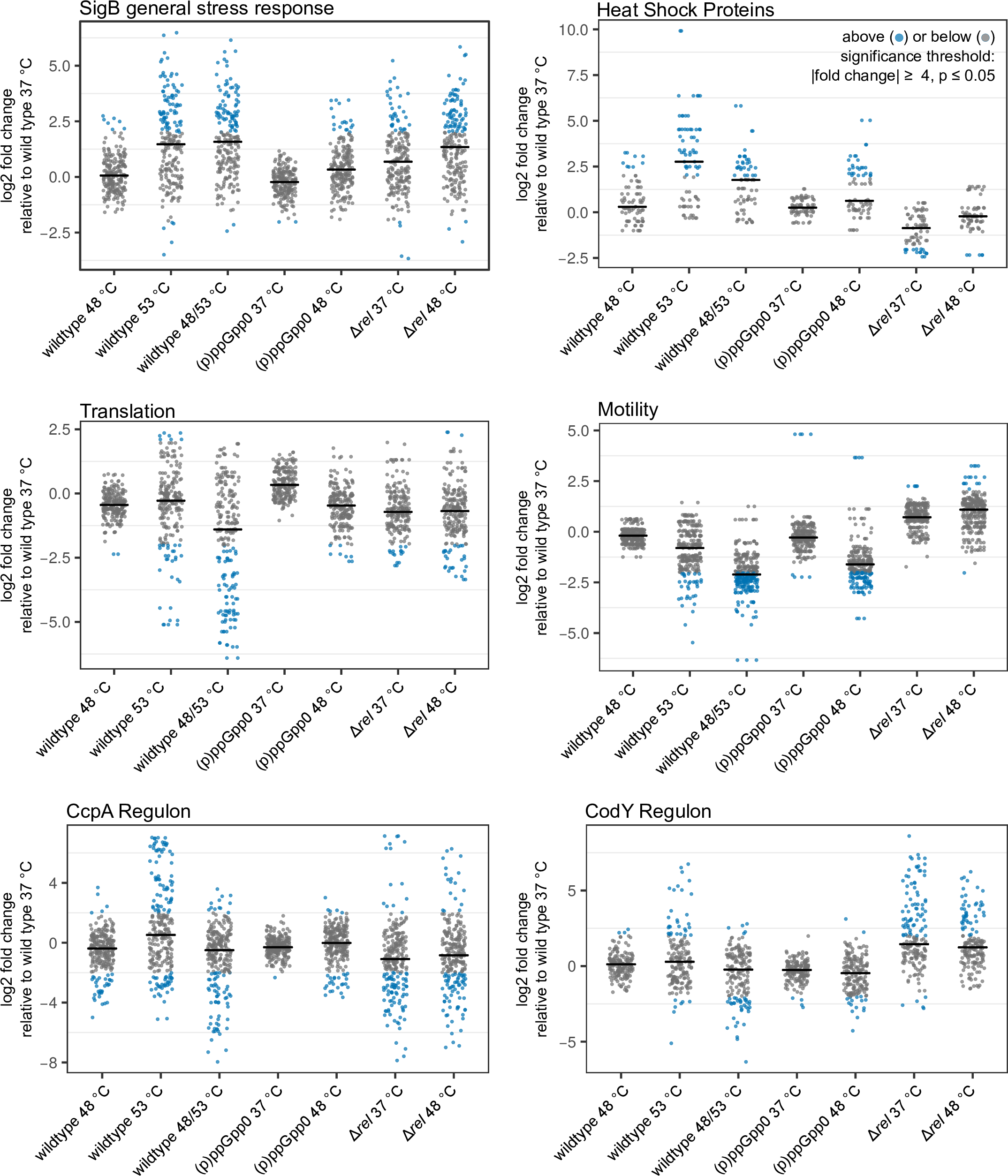

Supplement: S6 Fig — Points in the scatterplot represent log2-transformed up- or down-regulation of individual genes of the respective regulons relative to wild type cells at 37 °C. Blue/gray color indicates transcriptional changes above/below the significance threshold (see Materials and Methods). Horizontal bars represent the median expression changes of the whole gene set. (TIFF) [file pgen.1008275.s006.tiff]

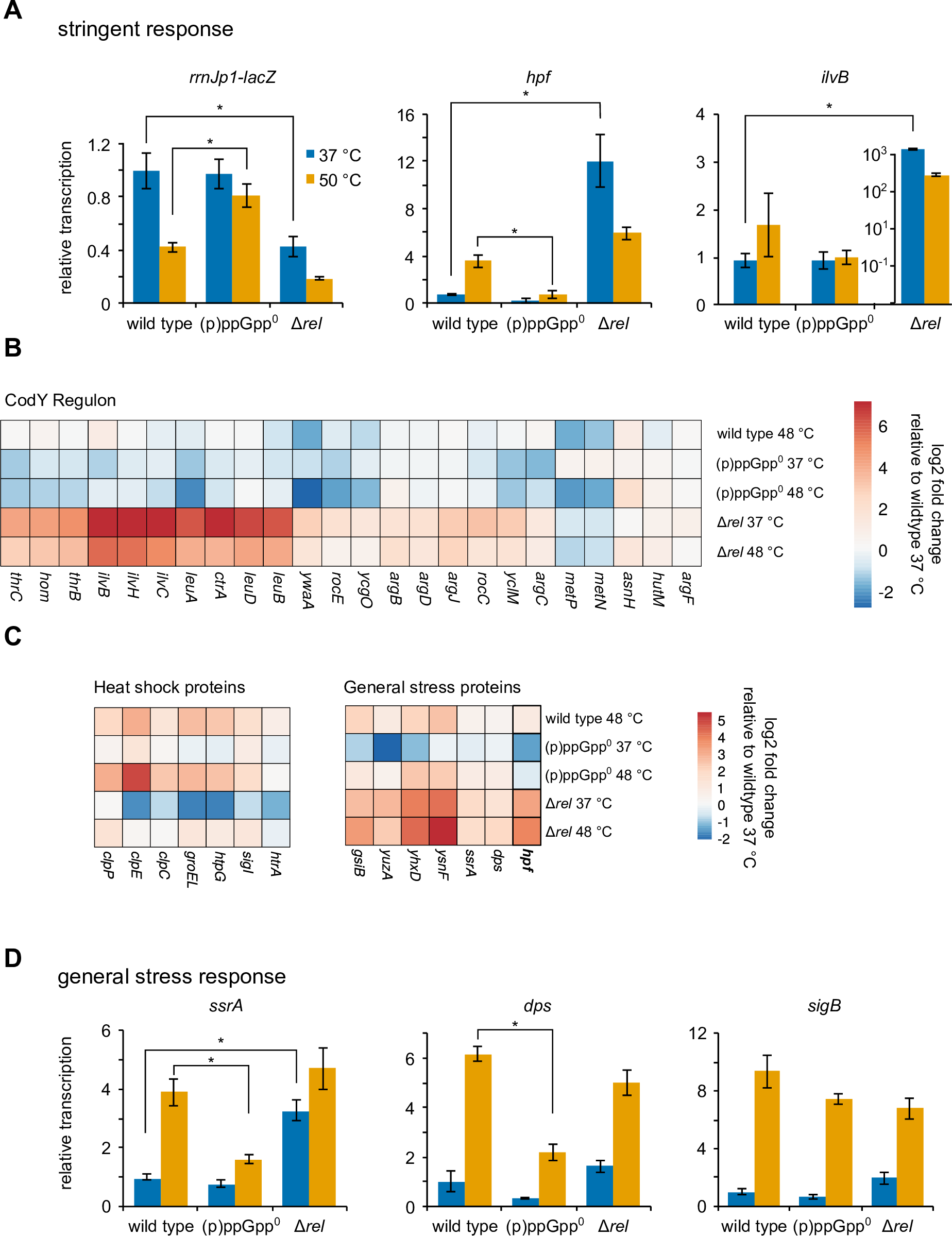

Supplement: S7 Fig — (A) Relative changes in the transcription of selected genes known to controlled by the stringent response during heat shock in wild type and (p)ppGpp° strains determined by RT-qPCR. Means and SEM of three replicates are shown. Asterisks indicate significance (p ≤ 0.05) according to Welch’s t-test. (B/C) Heatmap showing expression changes of selected transcripts during mild heat stress in wild type, (p)ppGpp° or Δrel cells. Values represent log2 fold changes of transcript levels relative to wild type cells at 37 °C. (D) Relative changes in the transcription of selected stress response genes during heat shock in wild type and (p)ppGpp° strains determined by RT-qPCR. Means and SEM of three replicates are shown. Asterisks indicate significance (p ≤ 0.05) according to Welch’s t-test. (TIFF) [file pgen.1008275.s007.tiff]

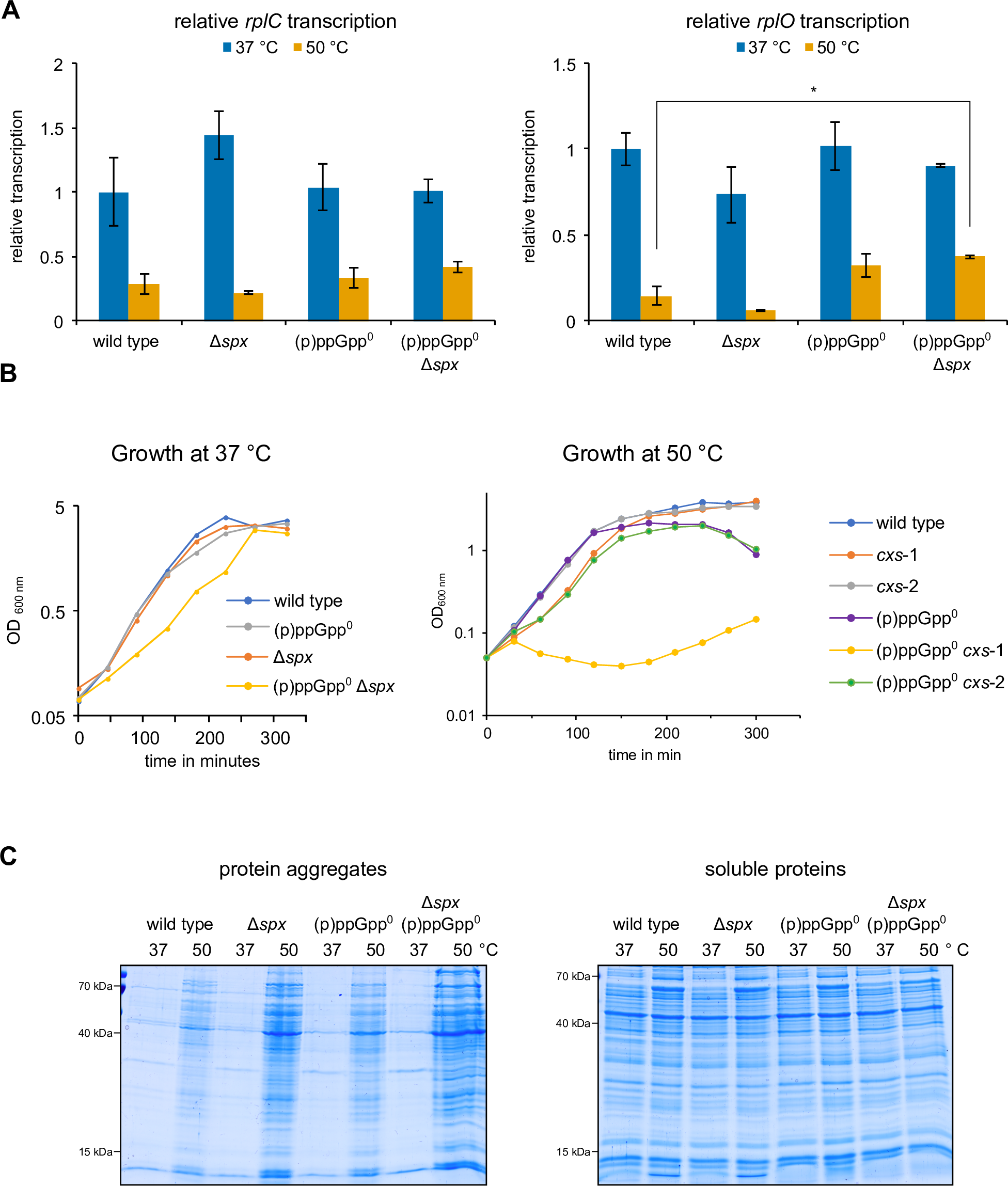

Supplement: S8 Fig — (A) RT-qPCR experiment showing the relative transcription of rplC and rplO in wild type (BHS220), Δspx (BHS222), (p)ppGpp° (BHS319) or (p)ppGpp° Δspx (BHS766) cells treated with or heat stress at 50 °C for 15 min. Means and SEM of three replicates are shown. Asterisks indicate significant changes (p ≤ 0.05) of transcript levels according to Welch’s t-test. (B) Growth of wild type, Δspx, (p)ppGpp° or (p)ppGpp° Δspx cells in LB medium at 37 °C (left) as well as growth of wild type, (p)ppGpp°, cxs-1, cxs-2, (p)ppGpp° cxs-1 or (p)ppGpp° cxs-2 cells in LB medium at 50 °C (right). (C) The fraction of aggregated proteins (left) or soluble proteins (right) in wild type, Δspx (BHS014), (p)ppGpp° (BHS214) or (p)ppGpp° Δspx (BHS766) cells treated with or heat stress at 50 °C for 15 min. (TIF) [file pgen.1008275.s008.tif]

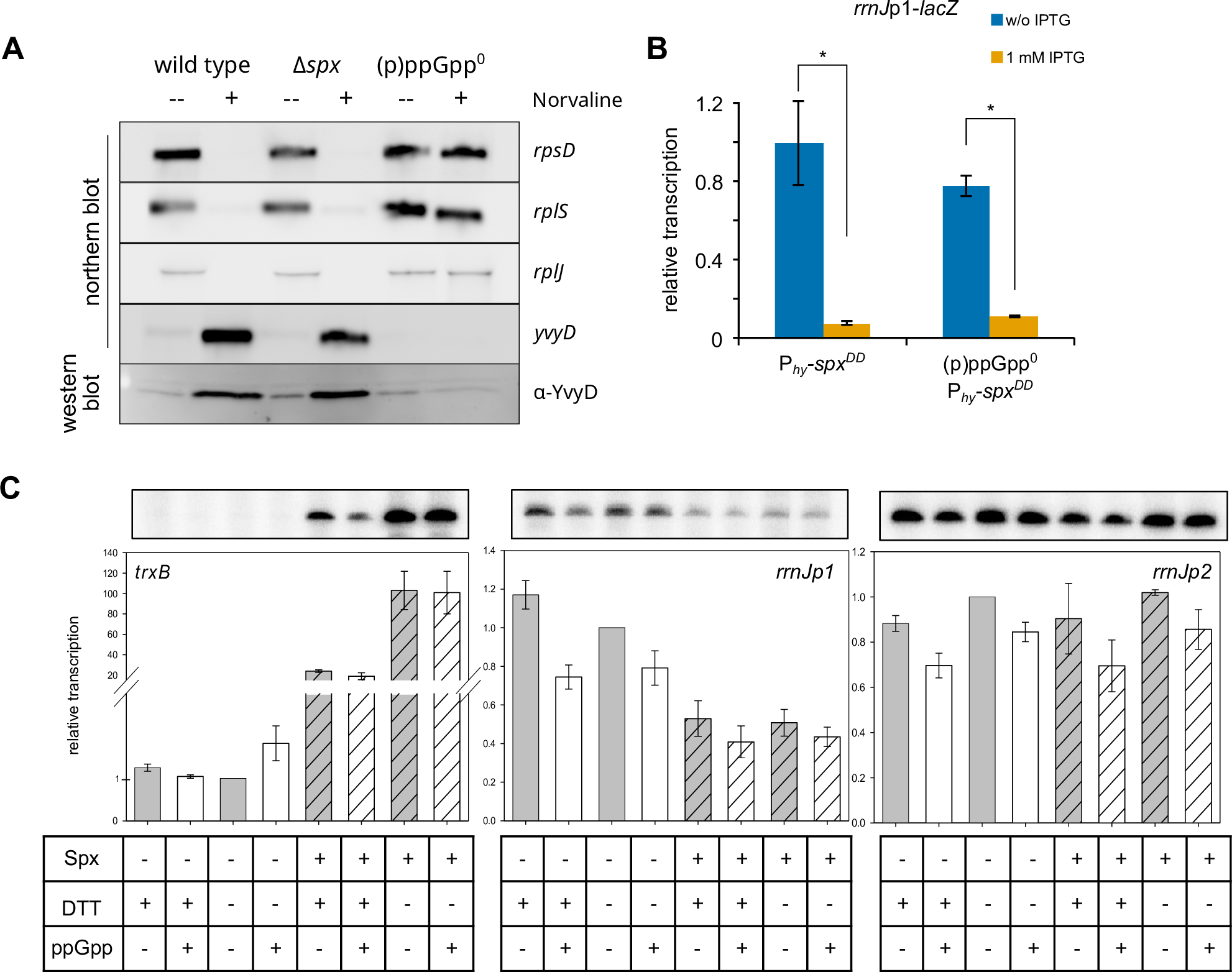

Supplement: S9 Fig — (A) Northern and western blot of wild type, Δspx (BHS014) or (p)ppGpp° (BHS214) strains treated with or without DL-norvaline. Cells were grown in minimal medium supplemented with 0.5% casamino acids to OD600 0.4. The medium was removed by centrifugation and the cells were resuspended in fresh medium with casamino acids (—) or 0.5 mg/ml DL-norvaline (+) and grown for 30 min. (B) Relative transcription of rrnJp1-lacZ with or without expression of spxDD with 1 mM IPTG for 30 min in the wild type or (p)ppGpp° background as determined by RT-qPCR. Means and SEM of three replicates are shown. Asterisks indicate significant changes (p ≤ 0.05) of transcript levels according to Welch’s t-test. (C) In vitro transcription experiments with selected promoters in the presence or absence of Spx or ppGpp under reducing (+ DTT) or oxidizing (- DTT) conditions. Means and SEM of three replicates and a representative autoradiogram are shown. (TIFF) [file pgen.1008275.s009.tiff]

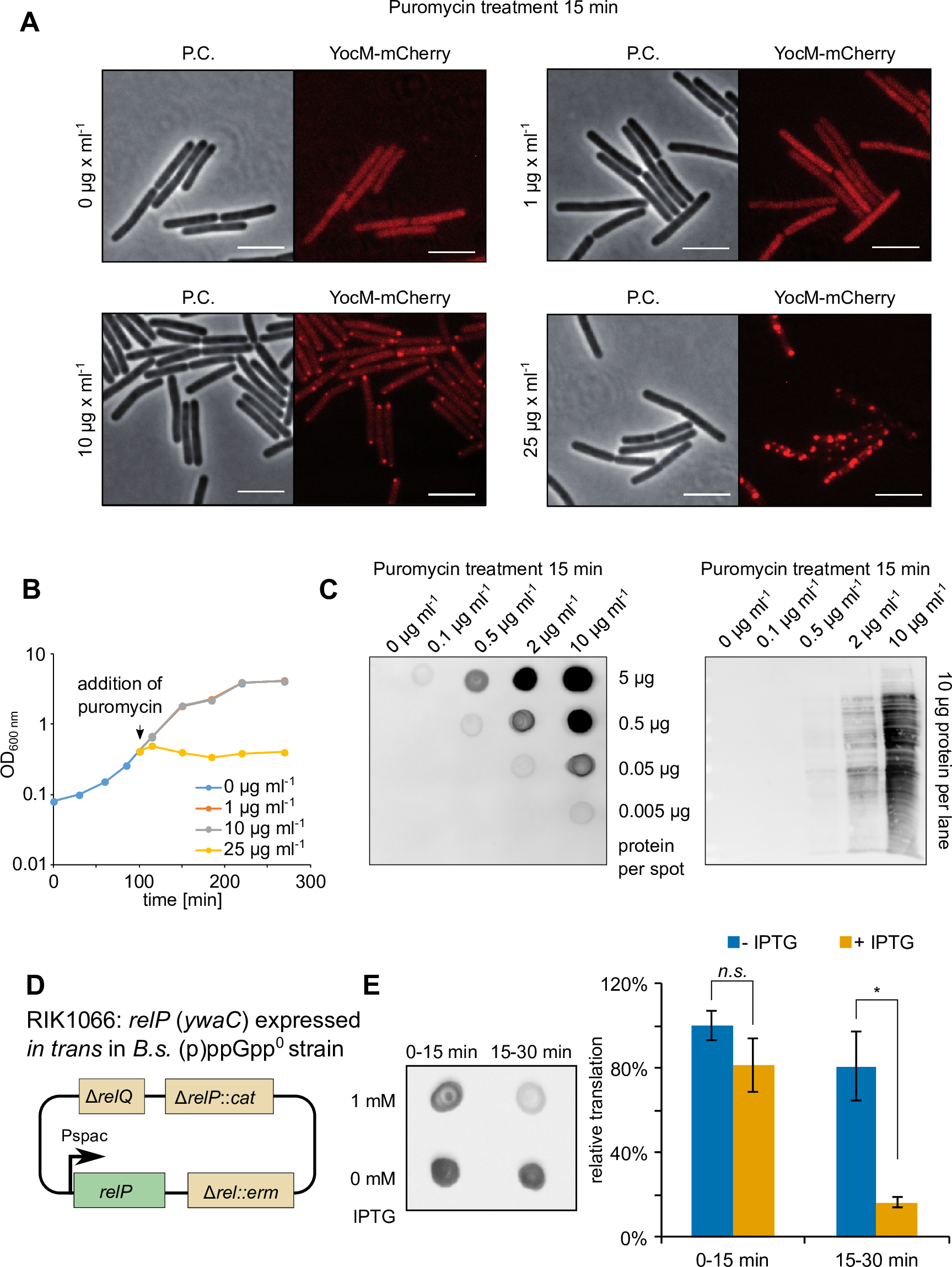

Supplement: S10 Fig — (A) Accumulation of subcellular protein aggregates (fluorescent spots) after the addition of puromycin visualized by YocM-mCherry. BIH369 cells were grown in LB + 0.5% xylose and treated with 1, 10 or 25 μg ml-1 puromycin or left untreated for 15 min. Phase contrast images (P.C.) and fluorescence images with RFP-filters (YocM-mCherry) are shown. (B) The effect of puromycin on growth. Wild type cells were grown in LB to the mid-exponential phase (OD600 0.4) and supplemented with puromycin at the indicated concentrations. (C) Dot blot or western blot of puromycin-labeled proteins. Exponentially growing cells grown in LB were treated with the indicated concentrations of puromycin for 15 min. (D) Outline of the genotypes of the RIK1066 strain, carrying an inducible copy of relP in the (p)ppGpp° background. (E) Relative puromycin incorporation in RIK1066 cells treated with or without 1 mM IPTG. Cells were incubated with 1 mg ml-1 puromycin for 15 min added directly to the medium after the addition of IPTG (0–15 min) or after 15 min (15–30 min), then harvested. One representative experiment and means and SEM from the quantification of three independent experiments are shown. Asterisks indicate significance (p ≤ 0.05) according to Welch’s t-test. (TIFF) [file pgen.1008275.s010.tiff]

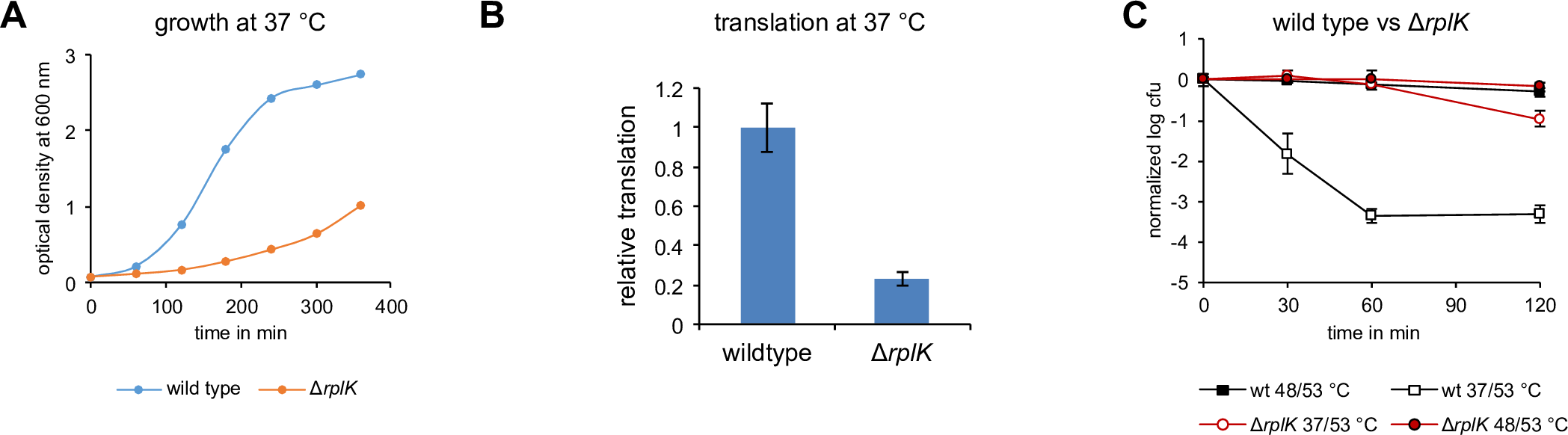

Supplement: S11 Fig — (A) Growth of wild type and ΔrplK (BHS859) cells. (B) Relative translation rates of wild type and ΔrplK (BHS859) cells at 37 °C. (C) Thermotolerance development and thermoresistance of ΔrplK (BHS859) cells. Means and standard error of three biological replicates are shown. (TIFF) [file pgen.1008275.s011.tiff]

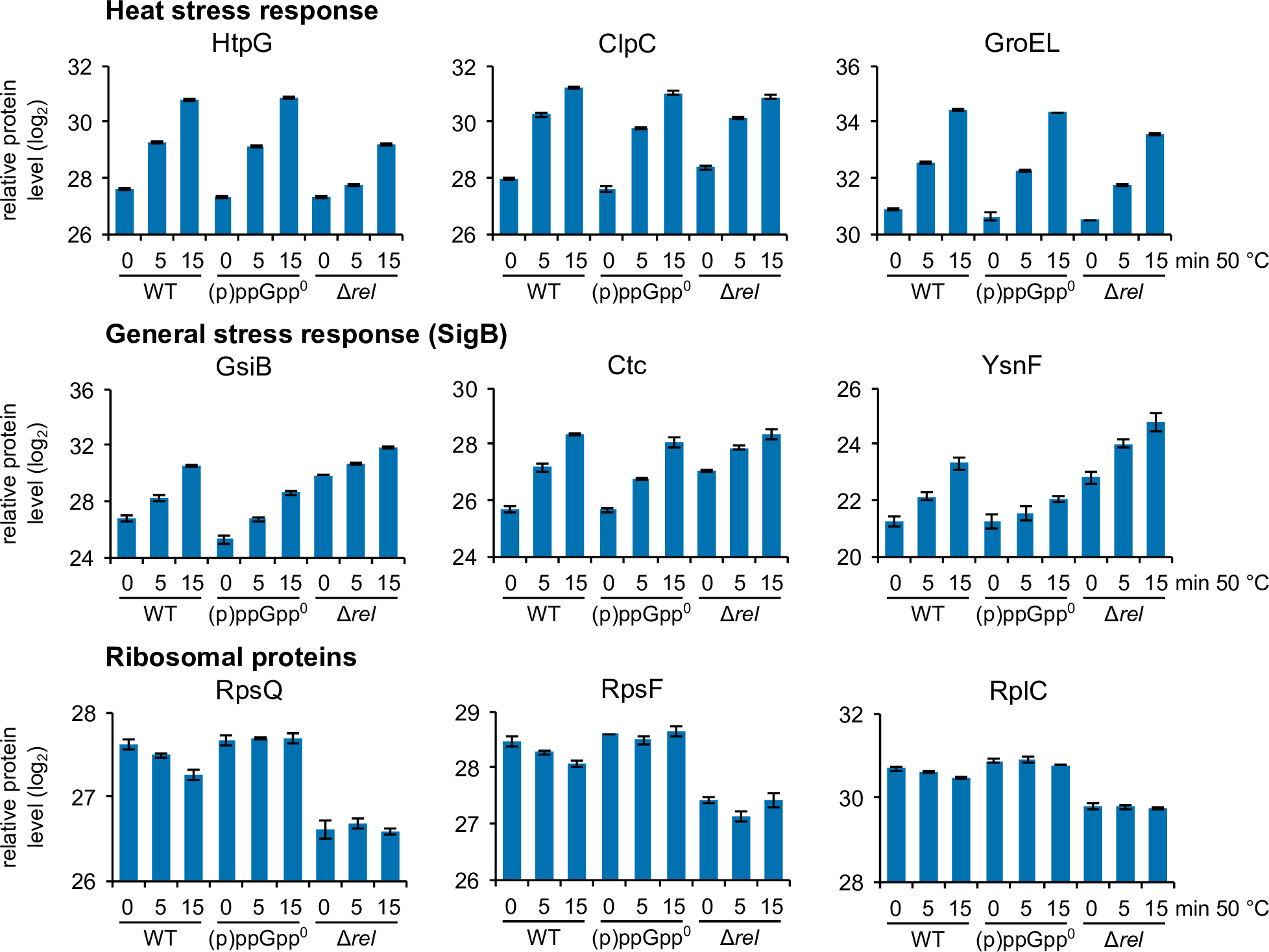

Supplement: S12 Fig — Levels of individual proteins in wild type and mutant strains with or without heat treatment (50 °C for 5 min or 15 min at 50 °C) relative to unstressed wild type cells. Means and standard error of three biological replicates are shown. (TIFF) [file pgen.1008275.s012.tiff]

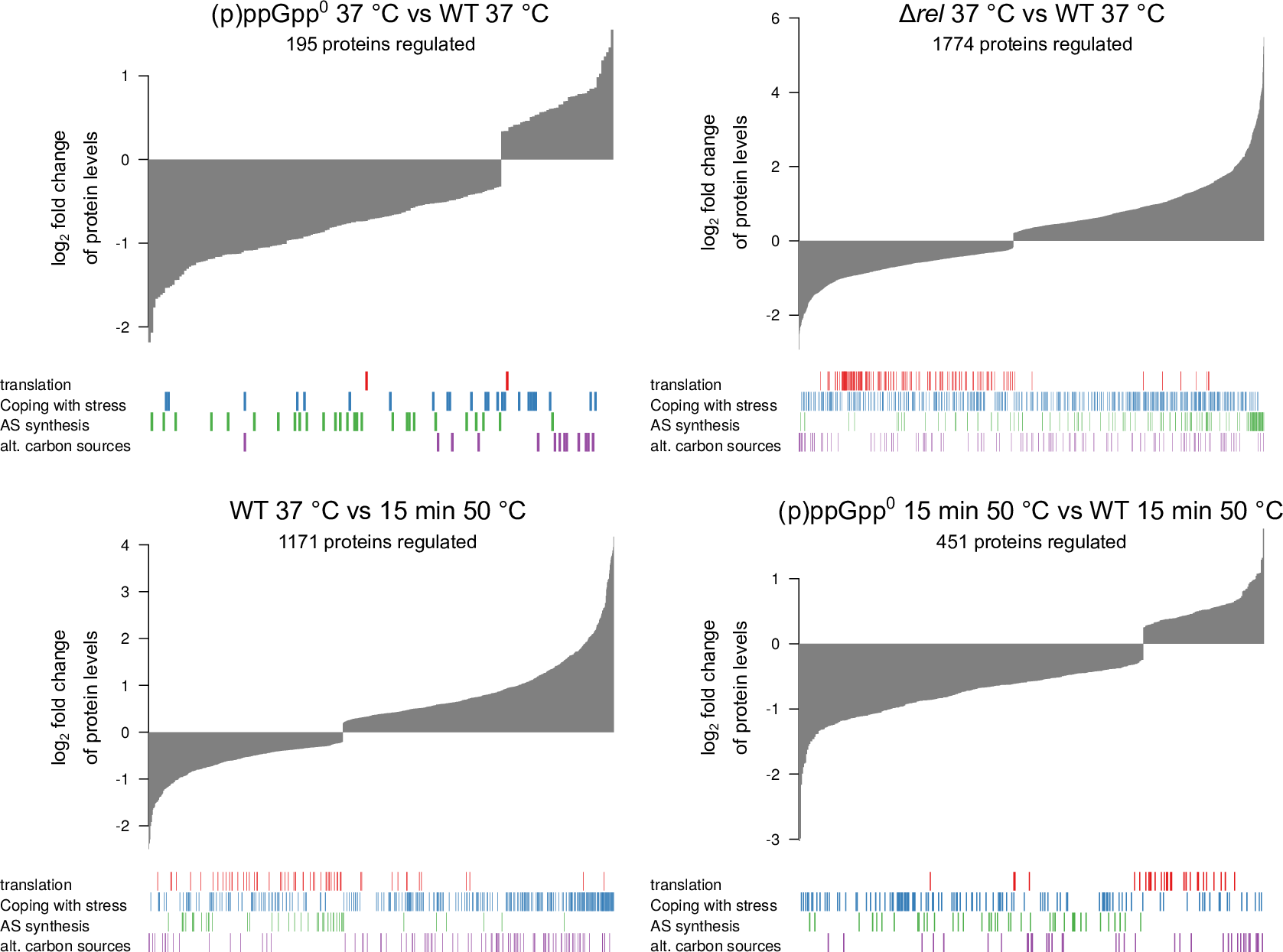

Supplement: S13 Fig — The distributions of all up- and down-regulated in wild type or mutant cells with or without heat treatment (15 min 50 °C). Bar tracks indicate the distribution of the respective functional groups. (TIFF) [file pgen.1008275.s013.tiff]

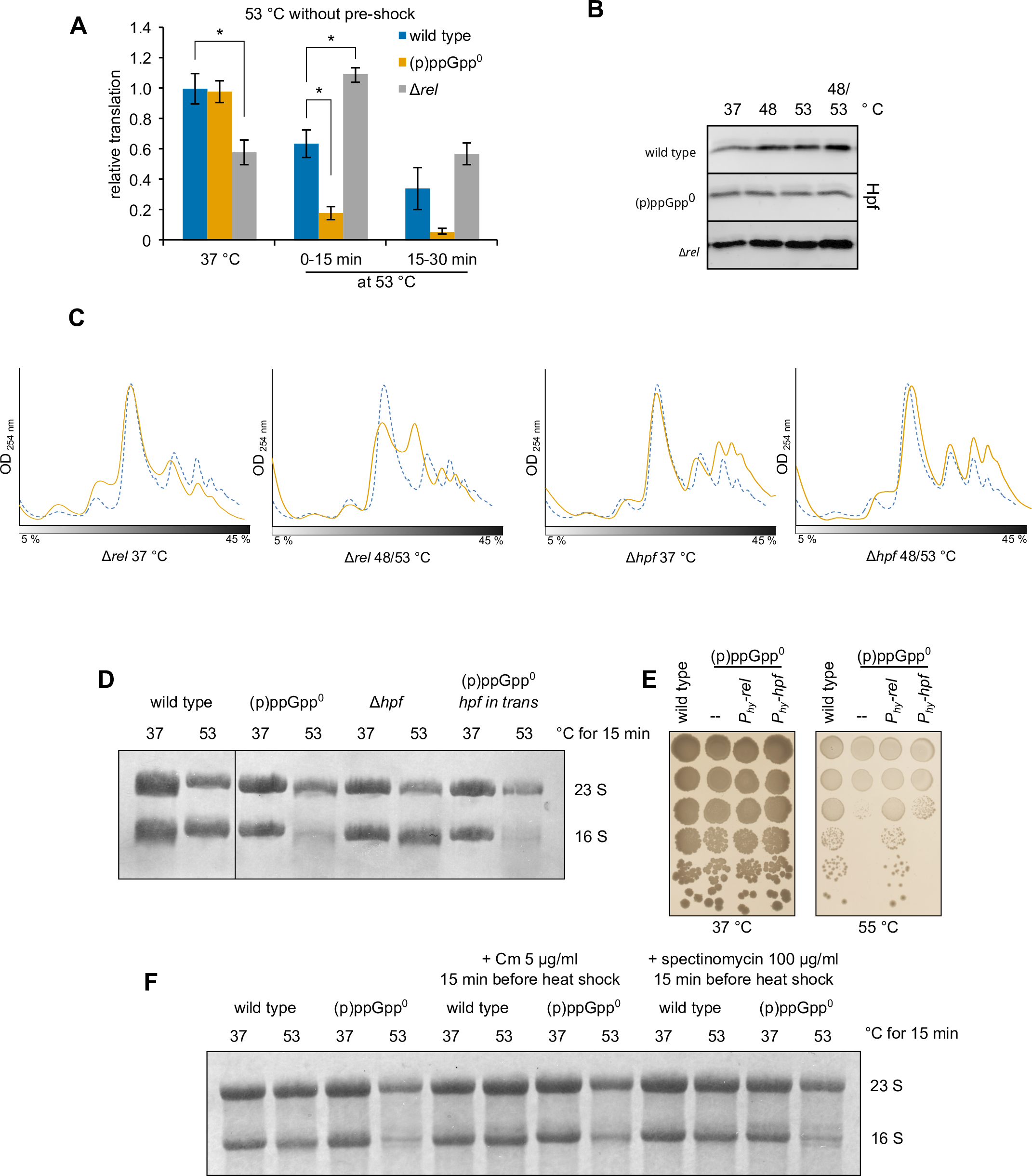

Supplement: S14 Fig — (A) Relative translation (puromycin incorporation) of wild type, (p)ppGpp° (BHS214) and Δrel (BHS126) strains during heat stress at 53 °C. 1 μg ml-1 puromycin was added for 15 min to the medium directly after (sample “0–15 min”) or 15 min (sample “15–30 min”) after the temperature upshift. Means and SEM of three independent experiments are shown. Asterisks indicate significance (p ≤ 0.05) according to Welch’s t-test. (B) Western blot showing Hpf levels during thermotolerance development in wild type, (p)ppGpp° (BHS214) or Δrel (BHS126) strains. Cells were heat shocked for 15 min each at the indicated temperature(s). (C) Sucrose gradient profiles of extracts from untreated (37 °C) or thermotolerant (48/53 °C for 15 min each) Δhpf (BHS008) or Δrel (BHS126) cells. The dashed blue line of untreated wild type cells is shown for reference. (D) Methylene blue stained membranes showing the integrity or degradation of rRNA. Wild type, (p)ppGpp° (BHS214) Δhpf (BHS008) or (p)ppGpp° Pspac-hpf (BHS626) cells were treated with or without heat shock at 53 °C for 15 min. 1 mM IPTG was added to the strains to induce the expression of hpf 15 min prior to heat shock. 2 μg total RNA was separated on denaturing agarose gels and blotted on nylon membranes. (E) Wild type, (p)ppGpp° (BHS214) (p)ppGpp° Pspac-rel (BHS622) or (p)ppGpp° Pspac-hpf (BHS626) were spotted on agar plates supplemented with 1 mM IPTG and incubated over night at 37 °C or 55 °C. (F) rRNA degradation after severe heat stress (53 °C) in wild type or (p)ppGpp° (BHS214) cells left untreated or treated with 5 μg ml-1 chloramphenicol or 100 μg ml-1 spectinomycin 15 min prior to the application of stress. 2 μg total RNA was separated on denaturing agarose gels and blotted on nylon membranes. (TIFF) [file pgen.1008275.s014.tiff]
