# Supplementary material for: The alarmones (p)ppGpp are part of the heat shock response of Bacillus subtilis
Source: PLoS Genet. 2020 Mar 16;16(3):e1008275. doi: 10.1371/journal.pgen.1008275 (PMC7098656; doi:10.1371/journal.pgen.1008275)
Supplement: S1 Table — This table lists all B. subtilis strains, plasmids and oligonucleotides used in this study. (DOCX) [file pgen.1008275.s015.docx]

# Table S1: List of plasmids, strains and oligonucleotides

## List of *B. subtilis* strains

| **strain** | **genotype** | **Source/construction** |
| --- | --- | --- |
| *wild type* | trpC2 | [1] |
| BNM111 | *trpC2 spx::kan* | [2] |
| BIH369 | *trpC2 lacA::P_xyl_-yocM-mCherry erm* | [3] |
| RIK900 | *trpC2 rel::erm* | [4] |
| RIK908 | *trpC2 relP::spec* | [4] |
| RIK909 | *trpC2 relQ::cat* | [4] |
| RIK1066 | *trpC2 ΔrelQ relP::cat rel::erm*  *aprE::Pspac-relP spc* | [5] |
| BKE01020 | *trpC2 ΔrplK::erm* | [6] |
| BHS008 | *hpf::kan* | [7] |
| BHS014 | *spx::kan* | [8] |
| BHS126 | *trpC2 rel::erm* | this work, Δ*rel* from RIK900 in wild type |
| BHS127 | *trpC2 relQ::cat* | this work, Δ*relQ* from RIK909 in wild type |
| BHS128 | *trpC2 relP::spec* | this work, Δ*relP* from RIK908 in wild type |
| BHS157 | *trpC2* *relQ*^E139V^ | this work, pMAD-relQE139V in wild type |
| BHS158 | *trpC2* *relP*^E154V^ | this work, pMAD-relPE154V in wild type |
| BHS204 | *trpC2* *relP*^E154V^ *relQ*^E139V^ | this work, pMAD-relQE139V in BHS158 |
| BHS214 | *trpC2* *relP*^E154V^ *relQ*^E139V^ *rel::erm* | this work, Δ*rel* in BHS204 |
| BHS220 | *trpC2 amyE::rrnJp1-lacZ cm* | [8] |
| BHS222 | *trpC2 spx::kan amyE::rrnJp1-lacZ cm* | [8] |
| BHS225 | *trpC2 amyE::rrnJp1-lacZ cm lacA::P_hy_-spx^DD^ erm* | [8] |
| BHS313 | *trpC2* *relP*^E154V^ *relQ*^E139V^ *amyE::rrnJp1-lacZ cm* | this work,  pDG268-rrnJp1 [8] in BHS204 |
| BHS319 | *trpC2* *relP*^E154V^ *relQ*^E139V^ *amyE::rrnJp1-lacZ cm rel::erm* | this work, Δ*rel* in BHS313 |
| BHS368 | *trpC2 rel::erm amyE::rrnJp1-lacZ cm* | this work, Δ*rel* in BHS220 |
| BHS610 | *trpC2 amyE::Phyperspank-relA_hyper_ spec* | this work, DGRM415 [9] in wild type |
| BHS611 | *trpC2 amyE::Phyperspank-relA_inactive_ spec* | this work, DGRM416 [9] in wild type |
| BHS618 | *trpC2* *relP*^E154V^ *relQ*^E139V^ *amyE::Phy-rel spec* | this work, pDR111-rel in BHS204 |
| BHS619 | *trpC2* *relP*^E154V^ *relQ*^E139V^ *amyE::Phy-rel^E324V^ spec* | this work, pDR111-relE324V in BHS204 |
| BHS620 | *trpC2* *relP*^E154V^ *relQ*^E139V^ *amyE::Phy-rel^H77A D78A^ spec* | this work, pDR111-relHDAA in BHS204 |
| BHS621 | *trpC2* *relP*^E154V^ *relQ*^E139V^ *amyE::Phy-hpf spec* | this work, pDR111-hpf in BHS204 |
| BHS622 | *trpC2* *relP*^E154V^ *relQ*^E139V^ *amyE::Phy-rel spec rel::erm* | this work, Δ*rel* in BHS618 |
| BHS624 | *trpC2* *relP*^E154V^ *relQ*^E139V^ *amyE::Phy-rel^E324V^ spec rel::erm* | this work, Δ*rel* in BHS619 |
| BHS625 | *trpC2* *relP*^E154V^ *relQ*^E139V^ *amyE::Phy-rel^H77A D78A^ spec rel::erm* | this work, Δ*rel* in BHS620 |
| BHS626 | *trpC2* *relP*^E154V^ *relQ*^E139V^ *amyE::Phy-hpf spec rel::erm* | this work, Δ*rel* in BHS621 |
| BHS709 | *trpC2 rel ^E324V^* | this work, pMAD-rel E324V in wild type |
| BHS755 | *trpC2* *relP*^E154V^ *relQ*^E139V^ *amyE::rrnJp1-lacZ cm spx::kan* | this work, Δ*spx* in BHS313 |
| BHS766 | *trpC2* *relP*^E154V^ *relQ*^E139V^ *amyE::rrnJp1-lacZ cm spx::kan rel::erm* | this work, Δ*rel* in BHS755 |
| BHS889 | *trpC2* *relP*^E154V^ *relQ*^E139V^ *amyE::rrnJp1-lacZ cm lacA::P_hy_-spx^DD^ spec* | this work, pBSII-spxDD-spec in BHS313 |
| BHS890 | *trpC2* *relP*^E154V^ *relQ*^E139V^ *amyE::rrnJp1-lacZ cm lacA::P_hy_-spx^DD^ spec* | this work, Δ*rel* in BHS890 |
| BHS942 | *trpC2* *relP*^E154V^ *relQ*^E139V^ *rpoA*^V260A^ | this work, PYZ38 [10] in BHS204 |
| BHS948 | *trpC2* *relP*^E154V^ *relQ*^E139V^ *rpoA*^V260A^ *amyE::rrnJp1-lacZ cm* | this work, pDG268-rrnJp1 [8] in BHS942 |
| BHS949 | *trpC2* *relP*^E154V^ *relQ*^E139V^ *rpoA*^V260A^ *amyE::rrnJp1-lacZ cm rel::erm* | this work, Δ*rel* in BHS948 |
| BHS952 | *trpC2* *relP*^E154V^ *relQ*^E139V^ *rpoA*^Y263C^ | this work, PYZ37 [10] in BHS204 |
| BHS953 | *trpC2* *relP*^E154V^ *relQ*^E139V^ *rpoA*^Y263C^ *amyE::rrnJp1-lacZ cm* | this work, pDG268-rrnJp1 [8] in BHS952 |
| BHS954 | *trpC2* *relP*^E154V^ *relQ*^E139V^ *rpoA*^Y263C^ *amyE::rrnJp1-lacZ cm rel::erm* | this work, Δ*rel* in BHS953 |

## List of plasmids

| **strain** | **Source or cloning primers** |
| --- | --- |
| pBSIIE | [11] |
| pDG268-rrnJp1 | [8] |
| pDR111 | [12] |
| pMAD | [13] |
| pDR111-hpf | [7] |
| pSN56 (pDR111-spxDD^)^ | [14] |
| pBSII-spxDD-spec | p203, p223, p288, p289 |
| pDR111-rel | p174, p274 |
| pDR111-rel E324V | p174, p274 |
| pDR111-rel H77A D78A | p174, p274 |
| pMAD-relQE139V | WS3-WS6 |
| pMAD-relPE154V | WS7-WS10 |
| pMAD-rel E324V | WS11, WS12, Ws13, WS14 |
| pMAD-rel H77A D78A | WS11, WS12, Ws15, WS16 |

## List of oligonucleotides used for cloning/sequencing

| **ID** | **name** | **Sequence** |
| --- | --- | --- |
| p68 | relP_seq | GAACCTTGCAGCAGACAGGG |
| p69 | relP_do_rev | CTATGACGCCAAACCTGTCG |
| p70 | relP_up_for | TTGCCTATGGATCCAGATCGC |
| p71 | relQ_up_for | CTGATACCTCTGAAAGCTGC |
| p72 | relQ_do_rev | CCTTATTGTAGGCTGTGCTG |
| p73 | relQ_seq | GCAAACTATGGAGAAGAAATGG |
| p74 | rel_up_for | GTGTGCTGTCTGTTGTGAGC |
| p75 | rel_do_rev | CAAAACGGCAAAACTGCTCG |
| p76 | rel_seq_for | TCTGCTCTTTACATCTTTCG |
| p77 | rel_seq_rev | CTGTATCATCGTGAGTGATG |
| p174 | SphI_rel_rev | ACATGCATGCTTAGTTCATGACGCGGCGCAC |
| p203 | pBS2E_for | TATACTAGTAGCGGCCGCTG |
| p219 | rel_intra_seq | TACGATTTGTTGGCTGTCCG |
| p223 | pDR111_rev | taACTAGT ATAATGGATTTCCTTACGCG |
| p274 | SalI_SD_rel_for | ACGCGTCGACTTGGGGGATGTATGATGGCGAACGAACAAGTATTG |
| p288 | NsiI_pBSIIE_rev | gatcATGCAT CCCTAGACTCTAGGACTCTC |
| p289 | NsiI_pdr111_for | gatcATGCAT ccctatgcaagggtttattg |
| WS01 | Bs*rel*-H420E-for | CTTACCGGATTGAATCTGAAATCGGC |
| WS02 | Bs*rel*-H420E-rev | GCCGATTTCAGATTCAATCCGGTAAG |
| WS03 | Bs*relQ*-flk1-EcoRI-F | TTAAGAATTCCCGCCCTGTAAATCTTATTT |
| WS04 | Bs*relQ* -flk2-NcoI-R | AATTCCATGGGTGCTGCCTGATGGAGTTGA |
| WS05 | Bs*relQ* -E139V-F | GAAAAGCATGTTCTCGTAGTAATACAGATCCGTACAC |
| WS06 | Bs*relQ* -E139V-R | GTGTACGGATCTGTATTACTACGAGAACATGCTTTTC |
| WS07 | Bs*relP* -EcoRI-F | TTAAGAATTCATGGATTTATCTGTAACAC |
| WS08 | Bs*relP* +fla-NcoI-R | TTAACCATGGAATCCAGCCGTACGGCTGC |
| WS09 | Bs*relP* -E154V-F | GTCAAAGCAGTAATTC |
| WS10 | Bs*relP* -E154V-R | GAATTACTGCTTTGAC |
| WS11 | BsRelA-EcoRI-F | TTAAGAATTCATGGCGAACGAACAAG |
| WS12 | BsRelA-Nco-R | TTAACCATGGTTAGTTCATGACGCGGCG |
| WS13 | Bs*rel*_E324V_for | GCGATCCGCTTGTAGTGCAGATCCG |
| WS14 | Bs*rel*_E324V_rev | CGGATCTGCACTACAAGCGGATCGC |
| WS15 | Bs*rel*_H77AD78A_for | GATTTTTGGCCGCTGTCGTGGAAGATAC |
| WS16 | Bs*rel*_H77AD78A_rev | GTATCTTCCACGACAGCGGCCAAAAATC |

## Oligonucleotides used for synthesis of RNA probes

| **ID** | **name** | **Sequence (T7 promoter underlined)** |
| --- | --- | --- |
| p162 | hpf_probe_for | CGTTAAAGGATCATGTCGAG |
| p163 | hpf_T7_rev | CTAATACGACTCACTATAGGGAGACGTCATTTCTGCGGTACACG |
| p297 | rpsD_probe_for | GGAATCTCTCTTAGCGGTAC |
| p299 | rpsD_T7_rev | CTAATACGACTCACTATAGGGAGAGCAAGTTCAGAACGCTCAGG |
| p300 | rplS_probe_for | CTTCGTACTGATCTTCCTGC |
| p302 | rplS_T7_rev | CTAATACGACTCACTATAGGGAGACTGATCTCTTTAATACGAGCC |
| p422 | rplJ_probe_for | ATGAGCAGCGCAATTGAAAC |
| p423 | rplJ_T7_rev | CTAATACGACTCACTATAGGGAGAGCGCCTTGTTCTTCCTTTTG |

## oligonucleotides used in RT-qPCR experiments

| **ID** | **name** | **Sequence** |
| --- | --- | --- |
| p585 | qPCR_lacZ_rev | cgtttcaccctgccataaag |
| p586 | qPCR_lacZ_for | ggaagatcaggatatgtggc |
| p595 | qPCR_rplC_for | TCCGGTAACTGTTATCGAGG |
| p596 | qPCR_rplC_rev | GACCAACTTCATACGCATCC |
| p601 | qPCR_hpf_for | AGGATCATGTCGAGAGGAAG |
| p602 | qPCR_hpf_rev | GCTTACGGATTTGACGTTCC |
| p605 | qPCR_23S_for | CTTTGATCCGGAGATTTCCG |
| p606 | qPCR_23S_rev | GTACAGAGTGTCCTACAACC |
| p642 | qPCR_sigB_for | AGCCTTATCCGTTGACCACA |
| p643 | qPCR_sigB_rev | GCGAGACGTGCATTTGAGAT |
| p648 | qPCR_ssrA_for | CGAGCTCTTCCTGACATTGC |
| p649 | qPCR_ssrA_rev | AACCCACGTCCAGAAACATC |
| p650 | qPCR_rplO_for | GTCGTGGTATTGGTTCTGGC |
| p651 | qPCR_rplO_rev | GTGACTTCCGTTCCTTCTGC |
| p664 | qPCR_dps_for | ACCGATTGCAACAATGAAGGA |
| p665 | qPCR_dps_rev | GTCCCCTGTTGTTTCGTCAC |
| p797 | qPCR_rbsC_for | GATGATTGTGTCCGGTGTCG |
| p798 | qPCR_rbsC_rev | GTAGTTTGTGCCAAGTCCGG |
| p799 | qPCR_ilvB_for | CATATCCTTCCCCGTCACGA |
| p800 | qPCR_ilvB_rev | GCTTCCTGAAATGCATCGCT |

# References

[1] J. Spizizen, “TRANSFORMATION OF BIOCHEMICALLY DEFICIENT STRAINS OF BACILLUS SUBTILIS BY DEOXYRIBONUCLEATE,” *Proc. Natl. Acad. Sci. U. S. A.*, vol. 44, no. 10, pp. 1072–1078, Oct. 1958.

[2] S. Runde *et al.*, “The role of thiol oxidative stress response in heat-induced protein aggregate formation during thermotolerance in *B* *acillus subtilis*: Thiol oxidation in protein aggregate formation,” *Mol. Microbiol.*, vol. 91, no. 5, pp. 1036–1052, Mar. 2014.

[3] I. Hantke, H. Schäfer, A. Janczikowski, and K. Turgay, “YocM a small heat shock protein can protect *Bacillus subtilis* cells during salt stress,” *Mol. Microbiol.*, vol. 111, no. 2, pp. 423–440, Feb. 2019.

[4] H. Nanamiya *et al.*, “Identification and functional analysis of novel (p)ppGpp synthetase genes in *Bacillus subtilis*,” *Mol. Microbiol.*, vol. 67, no. 2, pp. 291–304, Jan. 2008.

[5] K. Tagami *et al.*, “Expression of a small (p)ppGpp synthetase, RelP, in the (p)ppGpp(0) mutant of *Bacillus subtilis* triggers YvyD-dependent dimerization of ribosome,” *MicrobiologyOpen*, vol. 1, no. 2, pp. 115–134, Jun. 2012.

[6] B.-M. Koo *et al.*, “Construction and Analysis of Two Genome-Scale Deletion Libraries for Bacillus subtilis,” *Cell Syst.*, vol. 4, no. 3, pp. 291-305.e7, 22 2017.

[7] B. Beckert *et al.*, “Structure of the *Bacillus subtilis* hibernating 100S ribosome reveals the basis for 70S dimerization,” *EMBO J.*, vol. 36, no. 14, pp. 2061–2072, 14 2017.

[8] H. Schäfer *et al.*, “Spx, the central regulator of the heat and oxidative stress response in *B. subtilis*, can repress transcription of translation-related genes,” *Mol. Microbiol.*, vol. 111, no. 2, pp. 514–533, Feb. 2019.

[9] H. Nouri *et al.*, “Multiple links connect central carbon metabolism to DNA replication initiation and elongation in *Bacillus subtilis*,” *DNA Res. Int. J. Rapid Publ. Rep. Genes Genomes*, Sep. 2018.

[10] M. M. Nakano, Y. Zhu, J. Liu, D. Y. Reyes, H. Yoshikawa, and P. Zuber, “Mutations conferring amino acid residue substitutions in the carboxy-terminal domain of RNA polymerase alpha can suppress clpX and clpP with respect to developmentally regulated transcription in *Bacillus subtilis*,” *Mol. Microbiol.*, vol. 37, no. 4, pp. 869–884, Aug. 2000.

[11] J. Radeck *et al.*, “The Bacillus BioBrick Box: generation and evaluation of essential genetic building blocks for standardized work with *Bacillus subtilis*,” *J. Biol. Eng.*, vol. 7, no. 1, p. 29, Dec. 2013.

[12] S. Ben-Yehuda, D. Z. Rudner, and R. Losick, “RacA, a bacterial protein that anchors chromosomes to the cell poles,” *Science*, vol. 299, no. 5606, pp. 532–536, Jan. 2003.

[13] M. Arnaud, A. Chastanet, and M. Débarbouillé, “New vector for efficient allelic replacement in naturally nontransformable, low-GC-content, gram-positive bacteria,” *Appl. Environ. Microbiol.*, vol. 70, no. 11, pp. 6887–6891, Nov. 2004.

[14] S. Nakano, E. Küster-Schöck, A. D. Grossman, and P. Zuber, “Spx-dependent global transcriptional control is induced by thiol-specific oxidative stress in *Bacillus subtilis*,” *Proc. Natl. Acad. Sci. U. S. A.*, vol. 100, no. 23, pp. 13603–13608, Nov. 2003.
